# Association between sleep-disordered breathing and liver outcomes in steatotic liver disease: a systematic review and meta-analysis

**DOI:** 10.3389/fmed.2026.1884855

**Published:** 2026-09-08

**Authors:** Mohammad A. Alhajery, Abdulwahed Abdulaziz Alotay

**Affiliations:** Department of Internal Medicine, College of Medicine, Imam Mohammad Ibn Saud Islamic University (IMSIU), Riyadh, Saudi Arabia

**Keywords:** continuous positive airway pressure, hepatic fibrosis, intermittent hypoxia, metabolic dysfunction-associated steatohepatitis, metabolic dysfunction-associated steatotic liver disease, obstructive sleep apnea, sleep-disordered breathing, steatotic liver disease

## Abstract

**Background:**

Sleep-disordered breathing (SDB) including obstructive sleep apnea (OSA) has been linked to liver pathology development in patients with steatotic liver disease via pathways of intermittent hypoxia, oxidative stress, inflammation, and metabolic dysfunction. This systematic review and meta-analysis examined the relationship between SDB and liver outcomes in patients with steatotic liver disease (metabolic dysfunction-associated steatotic liver disease (MASLD) and metabolic dysfunction-associated steatohepatitis (MASH)).

**Methods:**

Review was performed according to PRISMA 2020 guidelines. The extensive search strategy was used for searching literature in databases PubMed/MEDLINE, Embase, Scopus, Web of Science Core Collection, Cochrane CENTRAL, CINAHL from inception to 15 May 2026. Bias assessment and grading of evidence were performed by design-specific criteria and using GRADE system correspondingly. The protocol for this review was registered in PROSPERO (CRD420261384244).

**Results:**

Twenty-two articles were included in the final analysis. Pooled prevalence of MASLD or hepatic steatosis among patients with SDB was 75.1% (95% CI 64.1–83.6%), however heterogeneity was moderate (I^2^ = 94.8%). The pooled prevalence of liver fibrosis or fibrosis risk was 13.9% (95% CI 6.0–29.0%, I^2^ = 96.8%). The presence of SDB or an increase in severity of OSA was significantly linked with higher probability of MASLD or steatosis (OR 3.62, 95% CI 1.59–8.25, I^2^ = 84.1%). The continuous positive airway pressure (CPAP) therapy showed reductions in alanine aminotransferase (mean difference −7.15 U/L, 95% CI −9.50 to −4.80; I^2^ = 35.9%) and aspartate aminotransferase (mean difference −3.38 U/L, 95% CI −4.45 to −2.30; I^2^ = 0.0%) levels. Based on qualitative synthesis, the measures of nocturnal hypoxemia were associated with liver damage stronger compared to apnea-hypopnea index (AHI). Meanwhile controlled CPAP trials failed to show consistent effects on structure of liver (steatosis, fibrosis, liver stiffness, and MASH-related parameters). Certainty of evidence ranged from low to very low for all outcomes.

**Conclusion:**

Sleep-disordered breathing was linked to a significant number of liver steatosis cases and higher odds of MASLD, while evidence on fibrosis outcomes was inconsistent. CPAP therapy showed improvements in biochemical liver injury markers, however, effect of CPAP on structure of liver remained inconclusive due to heterogeneity in study design, adherence to CPAP treatment and duration of follow-up. Further high-quality prospective trials are required to assess influence of SDB treatment on steatotic liver disease progression.

**Systematic review registration:**

PROSPERO, CRD420261384244.

## Introduction

Metabolic dysfunction-associated steatotic liver disease (MASLD) ranks among the most common chronic liver diseases throughout the world, and its burden will continue to grow as the rates of obesity, insulin resistance, type 2 diabetes mellitus, and metabolic syndrome increase (1). MASLD includes a wide clinicopathological range from pure hepatic steatosis to metabolic dysfunction-associated steatohepatitis (MASH), progressive fibrosis, cirrhosis, liver failure, and hepatocellular carcinoma (2). Epidemiological data suggest that MASLD accounts for up to one-third of the worldwide adult population; however, the disease burden is expected to grow, especially in low- and middle-income countries.

MASLD has additional clinical importance because it can progress to steatohepatitis and fibrosis of the liver. Among histological and non-invasive biomarkers, the stage of fibrosis has proved to be the best predictor of morbidity and all-cause mortality (3). Hence, recent scientific activity focuses on factors contributing to liver inflammation and fibrosis in patients with MASLD. Particularly, the conditions associated with oxidative stress, systemic inflammation, adipose tissue dysfunction, insulin resistance, and endothelial dysfunction may have a great influence on the progression of MASLD.

Sleep-disordered breathing (SDB) involves repeated episodes of partial or complete collapse of the upper airway during sleep leading to intermittent hypoxemia, sleep fragmentation, and sympathetic activation (4). OSA is prevalent among adults, and it often accompanies obesity, advanced age, male sex, hypertension, insulin resistance, dysglycemia, and other metabolic abnormalities that also lead to MASLD (5). The biological plausibility of the relationship between SDB and MASLD has to be acknowledged despite the difficulty in interpreting such a connection because of the overlapping of cardiometabolic risk factors.

The prevalence of OSA has risen significantly worldwide, and it currently accounts for about one in ten adults in many Western populations, although the actual number depends on the specific criteria, AHI threshold, and other population characteristics (6). Besides sharing the metabolic risk factors, experimental and translational evidence supports the direct contribution of SDB to the development of liver injury. Intermittent hypoxia contributes to oxidative stress, mitochondrial dysfunction, lipid peroxidation, activation of hypoxia-induced pathways, cytokines release, and liver stellate cell activation – processes involved in the formation of steatohepatitis and fibrosis (7). Furthermore, the hypoxemia at night contributes to insulin resistance, adipokine imbalance, free fatty acid flux, and low-grade inflammation that may cause additional damage to the liver (8). These mechanisms support the hypothesis that SDB can act as a modifier of the MASLD progression rather than a concomitant disease.

Clinical studies have shown the correlation between the severity of OSA, nocturnal oxygen desaturation, and MASLD progression indicators, such as increased levels of aminotransferases, liver steatosis detected by imaging, non-invasive indices of fibrosis, liver stiffness measured by elastography, and the features of steatohepatitis and fibrosis detected histologically (9). However, the clinical significance and reliability of the above correlations remain unclear due to the significant heterogeneity in the study populations, methods for the detection of SDB, liver disease evaluation, adjustment for metabolic confounders, and the definition of the outcomes. Studies that use polysomnography and biopsy of the liver to estimate the progression of MASLD are likely to be more reliable than those using only questionnaire, biomarker, and imaging-detected steatosis to detect the disorder.

The therapeutic implications of the above results remain uncertain, too. Although CPAP treatment was suggested as an intervention to decrease liver injury in OSA patients, the existing evidence is insufficient. Some studies have demonstrated the positive impact of CPAP on biochemical indicators of liver injury, while the results regarding the reduction of the extent of hepatic steatosis, inflammation, or fibrosis have been inconsistent and contradictory (10, 11). Moreover, the inconsistency is caused by the short term of the follow-up period, variations in the CPAP compliance, and different study designs.

Thus, taking into consideration the overlapping of metabolic risk factors, biological plausibility of the connection, and the insufficient clinical data, a comprehensive synthesis of the relevant literature is necessary. This is the aim of the present systematic review and meta-analysis evaluating the association between sleep-disordered breathing and liver-related outcomes in patients with steatotic liver disease, including MASLD and MASH.

## Materials and methods

### Review design and protocol

This systematic review was carried out according to the PRISMA 2020 guidelines (12). The review question was formulated using the PECOS framework. The protocol of the systematic review was registered in PROSPERO (CRD420261384244). The population consisted of human subjects diagnosed with steatotic liver disease, MASLD, MASH, MAFLD, or historical NAFLD/NASH phenotypes on the basis of clinical, radiological, transient elastography, histological, or biochemical criteria. The exposure of interest was sleep-disordered breathing (SDB) which includes obstructive sleep apnea (OSA), sleep apnea–hypopnea syndrome, intermittent nocturnal hypoxemia, oxygen desaturation, or other related polysomnographic parameters. Comparators were people without SDB or with less severe SDB. The outcomes of interest consisted of liver-related parameters, such as hepatic steatosis, levels of liver enzymes, activity of steatotic liver disease, fibrosis stage, liver stiffness, non-invasive fibrosis indices, cirrhosis-related outcomes, and changes in liver-related parameters after treatment of SDB. Eligible study designs were prospective and retrospective cohort studies, bariatric cohorts, sleep-clinic cohorts, hepatology cohorts, CPAP interventional studies randomized or not randomized, and longitudinal clinical studies reporting extractable liver-related outcomes.

### Inclusion and exclusion criteria

Eligible studies were those that examined the association between sleep-disordered breathing (SDB), obstructive sleep apnea (OSA), intermittent hypoxia, apnea–hypopnea index (AHI), oxygen desaturation index, or other related polysomnographic parameters and liver diseases such as MASLD, MASH, MAFLD, or similar steatotic liver diseases. Studies should have had extractable liver-related outcomes that include biochemical, radiological, elastographic, histological, or clinical endpoints.

Eligible study designs were cross-sectional studies, case–control studies, longitudinal cohort studies, and interventional studies studying the impact of CPAP or other SDB-related interventions. Studies that did not have extractable liver-related data were excluded from consideration. Other exclusion criteria were reviews, editorials, correspondence without primary data, conference abstracts lacking sufficient results, animal and *in vitro* studies, case reports, studies concerned solely with alcoholic liver disease or viral hepatitis lacking extractable steatotic liver disease outcomes, studies of obesity or metabolic syndrome lacking liver-specific outcomes, and studies where SDB was not considered independently.

### Database search protocol

Six databases, PubMed/MEDLINE, Embase, Scopus, Web of Science Core Collection, Cochrane CENTRAL, and CINAHL, were searched using terms for sleep-disordered breathing, contemporary steatotic liver disease terminology, historical NAFLD/NASH terminology, and liver-related outcomes. Database searches were performed from database inception to 15 May 2026, which was within 6 months of the expected manuscript submission time frame. In the context of this study, steatotic liver disease (SLD) is used as the umbrella terminology of contemporary times. MASLD is an abbreviation for metabolic dysfunction-associated steatotic liver disease, and MASH stands for metabolic dysfunction-associated steatohepatitis. Within this terminology, hepatic steatosis means the presence of excess hepatic fat determined by imaging, histological, or biochemical criteria, while MASLD is the clinical diagnosis in cases of co-occurrence of hepatic steatosis with at least one cardiometabolic risk factor. Since several studies included in this review reported the prevalence of steatosis without establishing the entire set of MASLD criteria, the findings are reported in this paper as “MASLD or steatosis” to take into account the difference in the definitions used in the source studies, rather than treating both of them as synonyms. Historical NAFLD/NASH terminology was maintained wherever it was necessary for reproducibility of the search, score names, and individual study results reporting.

Boolean operators, truncation, phrasal searching, proximity operators, and database-specific keywords were used. There was no criterion limiting the desired outcome in order to include studies that investigated liver endpoints either histologically, biochemically, or through ultrasound or other imaging techniques. All sources that were found through searching were loaded into the reference management software and duplicates were removed (Table 1).

**Table 1:** Search strings utilized across the databases.

Database	Search string
PubMed/MEDLINE	((“Sleep Apnea Syndromes”[Mesh] OR “sleep-disordered breathing”[tiab] OR “sleep disordered breathing”[tiab] OR “obstructive sleep apnea”[tiab] OR “obstructive sleep apnoea”[tiab] OR OSA[tiab] OR SAHS[tiab] OR “sleep apnea hypopnea syndrome”[tiab] OR “sleep apnoea hypopnoea syndrome”[tiab] OR “intermittent hypoxia”[tiab] OR “nocturnal hypoxemia”[tiab] OR “oxygen desaturation”[tiab] OR “apnea-hypopnea index”[tiab] OR AHI[tiab] OR CPAP[tiab]) AND (“Non-alcoholic Fatty Liver Disease”[Mesh] OR “Fatty Liver”[Mesh] OR NAFLD[tiab] OR NASH[tiab] OR “nonalcoholic fatty liver”[tiab] OR “non-alcoholic fatty liver”[tiab] OR “nonalcoholic steatohepatitis”[tiab] OR “non-alcoholic steatohepatitis”[tiab] OR MASLD[tiab] OR MAFLD[tiab] OR “metabolic dysfunction-associated steatotic liver disease”[tiab] OR “metabolic associated fatty liver disease”[tiab] OR “hepatic steatosis”[tiab] OR “liver steatosis”[tiab]) AND (“Liver”[Mesh] OR fibrosis[tiab] OR steatosis[tiab] OR “liver stiffness”[tiab] OR elastography[tiab] OR ALT[tiab] OR AST[tiab] OR transaminase*[tiab] OR “liver enzyme*”[tiab] OR histolog*[tiab] OR cirrhosis[tiab]))
Embase	(‘sleep disordered breathing’/exp. OR ‘sleep apnea syndrome’/exp. OR ‘obstructive sleep apnea syndrome’/exp. OR ‘sleep disordered breathing’:ti,ab OR ‘obstructive sleep apnea’:ti,ab OR ‘obstructive sleep apnoea’:ti,ab OR osa:ti,ab OR sahs:ti,ab OR ‘intermittent hypoxia’:ti,ab OR ‘nocturnal hypoxia’:ti,ab OR ‘oxygen desaturation’:ti,ab OR ‘apnea hypopnea index’:ti,ab OR ahi:ti,ab OR ‘continuous positive airway pressure’:ti,ab OR cpap:ti,ab) AND (‘nonalcoholic fatty liver disease’/exp. OR ‘non alcoholic steatohepatitis’/exp. OR ‘fatty liver’/exp. OR nafld:ti,ab OR nash:ti,ab OR ‘nonalcoholic fatty liver’:ti,ab OR ‘non-alcoholic fatty liver’:ti,ab OR ‘nonalcoholic steatohepatitis’:ti,ab OR ‘non-alcoholic steatohepatitis’:ti,ab OR masld:ti,ab OR mafld:ti,ab OR ‘hepatic steatosis’:ti,ab OR ‘metabolic dysfunction associated steatotic liver disease’:ti,ab) AND (‘liver function test’/exp. OR ‘liver fibrosis’/exp. OR ‘liver cirrhosis’/exp. OR fibrosis:ti,ab OR steatosis:ti,ab OR ‘liver stiffness’:ti,ab OR elastography:ti,ab OR aminotransferase*:ti,ab OR transaminase*:ti,ab OR alt:ti,ab OR ast:ti,ab OR histology:ti,ab)
Scopus	TITLE-ABS-KEY((“sleep-disordered breathing” OR “sleep disordered breathing” OR “obstructive sleep apnea” OR “obstructive sleep apnoea” OR OSA OR SAHS OR “sleep apnea hypopnea syndrome” OR “sleep apnoea hypopnoea syndrome” OR “intermittent hypoxia” OR “nocturnal hypoxia” OR “oxygen desaturation” OR “apnea-hypopnea index” OR AHI OR CPAP) AND (NAFLD OR NASH OR “nonalcoholic fatty liver” OR “non-alcoholic fatty liver” OR “nonalcoholic steatohepatitis” OR “non-alcoholic steatohepatitis” OR MASLD OR MAFLD OR “hepatic steatosis” OR “fatty liver disease”) AND (fibrosis OR steatosis OR “liver stiffness” OR elastography OR “liver enzyme*” OR aminotransferase* OR transaminase* OR ALT OR AST OR histolog* OR cirrhosis))
Web of Science Core Collection	TS = ((“sleep-disordered breathing” OR “sleep disordered breathing” OR “obstructive sleep apnea” OR “obstructive sleep apnoea” OR OSA OR SAHS OR “sleep apnea hypopnea syndrome” OR “sleep apnoea hypopnoea syndrome” OR “nocturnal intermittent hypoxia” OR “oxygen desaturation” OR “apnea-hypopnea index” OR CPAP) NEAR/10 (NAFLD OR NASH OR “nonalcoholic fatty liver” OR “non-alcoholic fatty liver” OR “nonalcoholic steatohepatitis” OR “non-alcoholic steatohepatitis” OR MASLD OR MAFLD OR “hepatic steatosis” OR “fatty liver”)) AND TS = (fibrosis OR steatosis OR “liver stiffness” OR elastography OR aminotransferase* OR transaminase* OR ALT OR AST OR histolog* OR cirrhosis OR “liver outcome*”)
Cochrane CENTRAL	((“sleep apnea” OR “sleep apnoea” OR “sleep-disordered breathing” OR “obstructive sleep apnea” OR “obstructive sleep apnoea” OR OSA OR “intermittent hypoxia” OR “oxygen desaturation” OR “apnea-hypopnea index” OR CPAP):ti,ab,kw) AND ((NAFLD OR NASH OR “nonalcoholic fatty liver” OR “non-alcoholic fatty liver” OR “nonalcoholic steatohepatitis” OR “non-alcoholic steatohepatitis” OR MASLD OR MAFLD OR “hepatic steatosis” OR “fatty liver”):ti,ab,kw) AND ((fibrosis OR steatosis OR elastography OR “liver stiffness” OR ALT OR AST OR aminotransferase OR transaminase OR histology OR cirrhosis):ti,ab,kw)
CINAHL	((MH “Sleep Apnea Syndromes+”) OR (TI “sleep-disordered breathing” OR AB “sleep-disordered breathing”) OR (TI “obstructive sleep apnea” OR AB “obstructive sleep apnea”) OR (TI “obstructive sleep apnoea” OR AB “obstructive sleep apnoea”) OR (TI OSA OR AB OSA) OR (TI “intermittent hypoxia” OR AB “intermittent hypoxia”) OR (TI “oxygen desaturation” OR AB “oxygen desaturation”) OR (TI “apnea-hypopnea index” OR AB “apnea-hypopnea index”) OR (TI CPAP OR AB CPAP)) AND ((MH “Fatty Liver+”) OR (TI NAFLD OR AB NAFLD) OR (TI NASH OR AB NASH) OR (TI “nonalcoholic fatty liver” OR AB “nonalcoholic fatty liver”) OR (TI “non-alcoholic fatty liver” OR AB “non-alcoholic fatty liver”) OR (TI “nonalcoholic steatohepatitis” OR AB “nonalcoholic steatohepatitis”) OR (TI MASLD OR AB MASLD) OR (TI MAFLD OR AB MAFLD) OR (TI “hepatic steatosis” OR AB “hepatic steatosis”)) AND ((MH “Liver Diseases+”) OR (TI fibrosis OR AB fibrosis) OR (TI steatosis OR AB steatosis) OR (TI “liver stiffness” OR AB “liver stiffness”) OR (TI elastography OR AB elastography) OR (TI “liver enzyme*” OR AB “liver enzyme*”) OR (TI ALT OR AB ALT) OR (TI AST OR AB AST) OR (TI histolog* OR AB histolog*))

### Data extraction protocol and selected data items

Independent extraction of data was done by two researchers using the same standard and pilot tested data extraction form. Study level data comprised authors’ names, year of publication, country, study design, study period, recruitment site, sample size, age, sex distribution, body mass index, presence of diabetes, and metabolic syndrome characteristics used in defining MASLD/MASH or relevant steatotic liver diseases phenotypes. Sleep disordered breathing (SDB) comprised diagnostic modality used, apnea-hypopnea index, oxygen desaturation index, nadir oxygen saturation, mean oxygen saturation, time of oxygen saturation below 90%, sleep fragmentation indices and treatments used mostly CPAP. Liver-related data comprised alanine transaminase, aspartate aminotransferase, gamma-glutamyl transferase, imaging-based liver steatosis, controlled attenuation parameter (CAP), MRI-proton-density fat fraction (MRI-PDFF), liver stiffness measurement (LSM), fibrosis scores, biopsy confirmed steatosis, components of activity score which include ballooning and lobular inflammation, MASH diagnosis, fibrosis stage, cirrhosis, and liver-related outcomes. Other data that were extracted comprised effect estimates, covariables, subgroups, follow-up period, missing data, and conflicts of interests.

### Risk of bias assessment protocol

The risk of bias assessment was done using specific tools for each design. Observational association studies including Cohort, Case–Control, and Cross Sectional designs were evaluated using Newcastle Ottawa Scale (NOS) adapted where necessary for non-cohort observational designs. Randomized CPAP trial studies were evaluated using Cochrane Risk of Bias 1.0 (ROB1) whereas Non-Randomized Intervention/Before-After treatment Cohort studies were appraised using ROBINS-I (risk of bias in non-randomized studies-interventions). The NOS risk of bias assessment comprised selection, comparability, and outcome/exposure assessment biases, including the representativeness of the exposed group, the appropriateness of the comparison group, reliability of OSA/SDB assessment, baseline definition of liver disease, and adjustment for important confounders including BMI/obesity, age, sex, diabetes mellitus, metabolic syndrome, dyslipidemia, alcohol consumption, and other causes of liver disease.

### GRADE certainty assessment

The certainty of evidence was judged independently by two reviewers following the GRADE approach (13) using the design-specific risk of bias judgments above. Evidence from randomized studies began at High certainty whereas from observational studies began at Low certainty. Factors considered for downgrading included risk of bias, inconsistency, indirectness, imprecision and publication bias. Residual confounding by obesity and metabolic disease, variability in SDB definitions, variability in liver outcomes definition, short follow-up period, and incomplete adjustment for cardiometabolic risk were particularly emphasized. Factors considered for upgrading were strong, consistent dose–response relations between SDB severity/hypoxia burden and liver outcome after adjusting for confounders. Judgments of certainty were summarized in the GRADE evidence profiles and summary of findings tables.

### Meta-analysis protocol

The meta-analysis design and performance were undertaken using MetaAnalysisOnline.com (Meta-Analysis Software) (14). Data sets were combined if there were at least two sets of data which were comparable clinically and methodologically in terms of their liver-related outcome or estimates that can be included in meta-analysis. Dichotomous outcomes such as presence of NAFLD, NASH, fibrosis and advanced fibrosis were analyzed using Odds ratios, risk ratios and adjusted effects. Continuous outcomes such as alanine transaminase, aspartate aminotransferase, liver stiffness, controlled attenuation parameter and fibrosis score were analyzed using mean differences or standardized mean differences according to their scale consistency. Adjusted effects would be analyzed preferentially using the generic inverse variance method after log transformation whenever available. The application of random effect model is appropriate due to the anticipated heterogeneity across population, SDB severity, diagnostics and definition of liver-related outcomes.

## Results

**Figure 1:**
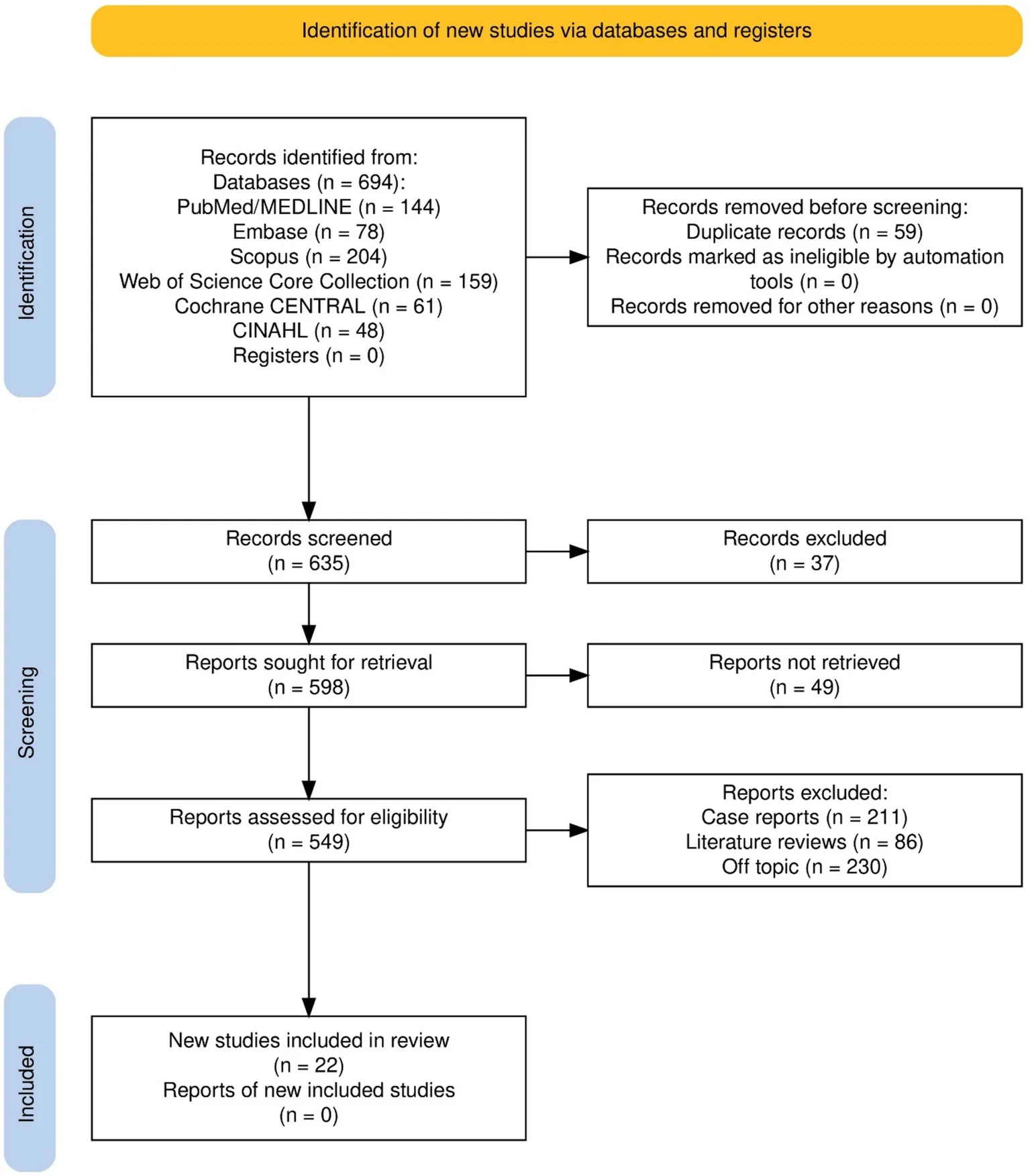
Study selection process as per PRISMA 2020 guidelines.

The selection process of the research included 694 articles which were identified by searching in six different databases: PubMed/MEDLINE (144 articles), Embase (78), Scopus (204), Web of Science Core Collection (159), Cochrane CENTRAL (61) and CINAHL (48) as indicated in Figure 1 below. After exclusion of 59 duplicate articles, 635 studies were then screened and 37 of them were excluded using information given in the title and abstract. 598 full texts were obtained and 49 could not be retrieved as the full text was not available. Thus, 549 articles were considered for the eligibility screening. In consequence, 527 articles were excluded because those were case reports (*n* = 211), literature reviews (*n* = 86), and other non-relevant articles (*n* = 230). In this way, 22 articles (15–36) were selected for the systematic review.

### Risk of bias observations across the included studies

There was an observation of relatively low to moderate risk of bias assessment across the included studies. No one study was assessed to have a high risk of bias. In particular, an overall low risk of bias was identified for the studies by Jouët et al. (15), Weingarten et al. (21), Corey et al. (22), Agrawal et al. (23), Toyama et al. (25), Sundaram et al. (28), Zhang et al. (29), Ng et al. (31), Lian et al. (33), Gul et al. (35), and Trzepizur et al. (36). Overall, those studies were associated with the appropriate case ascertainment, reliable or correctly defined OSA/SDB assessment, adequate liver outcome measurement, and proper adjustment for potential biases. The overall moderate risk of bias was identified in the studies by Kallwitz et al. (16), Polotsky et al. (17), Shpirer et al. (18), Ulitsky et al. (19), Byrne et al. (20), Jullian-Desayes et al. (24), Chen et al. (26), Kim et al. (27), Hirono et al. (30), Jawa et al. (32), and Landete et al. (34). Those studies were associated with the retrospective design, relatively small sample sizes, biochemical or non-invasive liver outcome measurement, limited adjustment of confounders, or unclear missing data description.

### Study population characteristics

Most of the studies involved were recruited from the bariatric, sleep clinic, hepatology, respiratory, and biopsy-confirmed MASLD patient groups (Table 2). Bariatric patients constituted a relatively higher percentage of evidence in the early years, where the body fat of the patients was extremely high in Jouët et al. (15), Kallwitz et al. (16), Polotsky et al. (17), Weingarten et al. (21), Corey et al. (22), and Zhang et al. (29), where mean BMI ranged between 37 to 55 kg/m^2^. Further studies included more sources for the population through addition of non-obese adults (33), pediatric biopsy-confirmed MASLD patients (28), and biopsy-confirmed suspected MASLD patients (36).

**Table 2:** Demographic characteristics of the included studies.

Study ID	Year	Study design	Country	Setting/source	Population	Sample size	Age/sex profile	BMI/adiposity profile	OSA/SDB presence
Jouët et al. (15)	2007	Prospective observational bariatric study	France	Bariatric surgery unit	Consecutive morbidly obese patients requiring bariatric surgery	62	38.5 ± 11.0 years; 54 female/8 male	Mean BMI 47.8 ± 8.4 kg/m^2^	OSA defined by AHI > 10/h; OSA in 84.7%
Kallwitz et al. (16)	2007	Retrospective bariatric cohort	USA	Obesity surgery cohort	Patients with sleep study before surgery and liver biopsy at surgery	85	Mean age 44 ± 11 years; 72% female	Mean BMI 55 ± 11 kg/m^2^	Clinically significant OSA defined as AHI ≥ 15; OSA in 51%
Polotsky et al. (17)	2009	Prospectively recruited severe-obesity cohort	USA	Bariatric surgery clinic	Severely obese adults without known OSA, diabetes, or liver disease	90; biopsy subset *n* = 20	41.1 ± 9.5 years; 75 women/15 men	Mean BMI 49.0 ± 7.9 kg/m^2^	OSA defined by RDI ≥ 5/h; OSA in 81.1%
Shpirer et al. (18)	2010	Prospective non-randomized CPAP cohort	Israel	Sleep laboratory	OSA patients assessed for liver fat by CT	47	54.7 ± 7.9 years; 84.8% male	BMI was higher in the low liver attenuation index (LAI) subgroup; LAI denotes CT liver attenuation index, with lower values indicating greater hepatic fat content.	OSA defined by AHI ≥ 5/h; CPAP follow-up in low-LAI patients
Ulitsky et al. (19)	2010	Retrospective bariatric prediction cohort	USA	Bariatric surgery records	Morbidly obese patients undergoing surgery with intraoperative liver biopsy	253	Mean age 43.2 years; 86.6% female	Morbid obesity; BMI not significantly different by MASH status	OSA defined by documented diagnosis and/or diagnostic sleep study
Byrne et al. (20)	2012	Retrospective sleep-lab cohort	USA	Hospital-based sleep laboratory	Consecutive patients referred for suspected OSA with liver enzymes near sleep study	53	60% male; OSA group 63.6 years vs. non-OSA 67.7 years	OSA group BMI 33.3 vs. non-OSA 30.6 kg/m^2^	OSA defined as AHI > 10
Weingarten et al. (21)	2012	Retrospective bariatric cohort	USA	Mayo Clinic bariatric surgery cohort	Medically complicated obese patients with PSG and intraoperative liver biopsy	218	Mean age 48.2 ± 10.8 years; 77.5% female	Mean BMI 47.9 ± 9.1 kg/m^2^	No/mild OSA AHI ≤ 15 vs. moderate/severe OSA AHI ≥ 16
Corey et al. (22)	2013	Bariatric cohort; retrospective OSA ascertainment	USA	Weight-loss surgery cohort	Obese adults undergoing bariatric surgery with liver biopsy	159	Normal liver group 40.3 years; MASLD group 47.8 years	Obese bariatric population; BMI not significantly different by liver status	OSA based on medical-record diagnosis
Agrawal et al. (23)	2015	Consecutive clinic-based observational cohort	India	Hepatology and chest clinics	MASLD patients screened for OSA and OSA patients screened for MASLD	100 MASLD + 23 OSA	MASLD group 41 ± 11 years; OSA group 46 ± 12 years	Indian MASLD/OSA population; less obese than Western bariatric cohorts	OSA diagnosed by PSG; AHI ≥ 5
Jullian-Desayes et al. (24)	2016	Randomized sham-controlled CPAP trial analysis	France/Switzerland	Sleep laboratories; pooled data from 3 RCTs	Moderate-to-severe OSA patients enrolled in CPAP vs. sham CPAP trials	103 analysed	CPAP group median 57 years, 84.3% men; sham group median 57 years, 73.1% men	BMI about 28 kg/m^2^	AHI > 15/h; randomized to effective vs. sham CPAP
Toyama et al. (25)	2017/2018	Long-term non-randomized CPAP cohort	Japan	Clinical OSA cohort with abdominal CT	Male OSA patients with abdominal obesity assessed before and after CPAP	61	Male only	Abdominal obesity; 25 had fatty liver at baseline	OSA patients treated with CPAP; mean therapy duration 31 months
Chen et al. (26)	2018	Observational study with non-randomized CPAP follow-up subgroup	China	Sleep laboratory	Consecutive adults referred for snoring/suspected OSA	160; CPAP subgroup *n* = 28	42.64 ± 12.06 years; 139 male/21 female	Mean BMI 28.00 ± 4.35 kg/m^2^	PSG-defined OSA; control *n* = 30, moderate OSA *n* = 42, severe OSA *n* = 88
Kim et al. (27)	2018	Retrospective CPAP-treated cohort	USA	Stanford institutional database	Adults with OSA treated with CPAP and available ALT before/after therapy	351	Mean age 57.6 years; 59.3% male	Mixed obesity classes; BMI less favorable in severe OSA	PSG-defined OSA; CPAP adherence classified as poor/partial/good
Sundaram et al. (28)	2018	Pediatric prospective before–after CPAP pilot study	USA	Pediatric liver/sleep cohort	Children/adolescents with biopsy-proven MASLD and OSA/nocturnal hypoxia	9	11.5 ± 1.2 years; pediatric cohort	BMI 29.5 ± 3.8 kg/m^2^	PSG-confirmed OSA/nocturnal hypoxia treated with home CPAP
Zhang et al. (29)	2020	Retrospective bariatric surgery follow-up cohort	China	Metabolic and bariatric surgery center	Obese patients with OSA undergoing metabolic bariatric surgery	181 baseline; 127 completed 6-month follow-up	Mild–moderate OSA: 30 ± 9 years; severe OSA: 34 ± 10 years	BMI 37.2 ± 6.4 vs. 41.0 ± 6.9 kg/m^2^ in mild–moderate vs. severe OSA	PSG-defined OSA; mild–moderate *n* = 80, severe *n* = 101
Hirono et al. (30)	2021	Prospective non-randomized CPAP cohort	Japan	Sleep laboratory/internal medicine setting	Moderate-to-severe OSA patients indicated for CPAP	123 enrolled; 70 analysed; MASLD subgroup *n* = 50	Adult cohort; detailed subgroup values reported in tables	MASLD and normal liver groups by ultrasound	PSG-defined moderate-to-severe OSA; all treated with auto-CPAP for 6 months
Ng et al. (31)	2022/2021	Randomized controlled trial; abridged secondary publication	Hong Kong	Hepatology and respiratory clinic	Adults aged 20–80 years with biopsy-proven MASLD and OSA	120 randomized; 60 auto-CPAP, 60 subtherapeutic CPAP	Auto-CPAP 54.62 ± 10.37 years; subtherapeutic 54.75 ± 9.71 years	BMI 28.62 ± 4.47 vs. 28.72 ± 4.22 kg/m^2^	Home sleep test; REI ≥ 5/h; auto-CPAP vs. subtherapeutic CPAP
Jawa et al. (32)	2021	Retrospective OSA cohort	Saudi Arabia	Sleep Medicine and Research Center	Confirmed OSA patients who underwent abdominal ultrasonography	133	49.8 ± 15.1 years; 57.1% female	Mean BMI 37.3 ± 14.5 kg/m^2^	PSG-confirmed OSA; mild 37.9%, moderate 29.6%, severe 32.6%
Lian et al. (33)	2022	Retrospective non-obese sleep-center cohort	China	Sleep Respiratory Disease Research Center	Consecutive non-obese adults with suspected sleep apnea	410	Mean age 53.0 ± 14.4 years; 79% male	Non-obese Chinese population; median BMI 25.3 kg/m^2^	PSG-defined simple snoring, mild–moderate, severe, and very severe OSA
Landete et al. (34)	2022	Prospective cohort study	Spain	Pneumology (pulmonology) outpatient clinic	OSA patients and normal lung function controls	196; OSA *n* = 153, controls *n* = 43	Men predominated in OSA group	Mixed BMI; obesity more frequent in OSA group but not significant	Polygraphy-defined OSA; AHI, ODI, and Tc90% assessed
Gul et al. (35)	2026	Prospective observational clinical study	Türkiye	Respiratory disease outpatient clinic	Adults evaluated for suspected OSA	400	Adults ≥18 years; full demographic table available in article	Mixed BMI; obesity/metabolic syndrome assessed	Full-night PSG; simple snoring, mild, moderate, severe OSA
Trzepizur et al. (36)	2026	Prospective multicentric biopsy-based cohort	France	Patients with suspected MASLD referred for liver biopsy	Suspected MASLD patients undergoing percutaneous liver biopsy and PSG	97	Adult cohort of consecutive patients with suspected MASLD referred for liver biopsy and PSG; detailed age/sex values were not extractable from the supplied manuscript.	Metabolic-risk MASLD cohort with high obesity/metabolic syndrome burden reported.	In-lab PSG; moderate–severe OSA defined as AHI ≥ 15 events/h

### Number of subjects and distribution of exposure

The sample size ranged from 9 subjects for the pediatric CPAP pilot study (28) to 410 non-obese suspected sleep apnea patients in Lian et al. (33) and 400 suspected OSA patients in Gul et al. (35). The OSA occurrence was quite high in those studies conducted on bariatric or MASLD patients, such as 84.7% in Jouët et al. (15) and 81.1% in Polotsky et al. (17). The composition of the population provided clear evidence that metabolic risks were enriched, with obesity, diabetes, and metabolic syndrome as key covariates in interpretation.

### Definition and assessment of OSA/SDB

In the studies under consideration, OSA/SDB was well-defined, and the disease diagnosis and severity were determined by objective sleep testing, namely, polysomnography, home sleep testing, or cardiorespiratory polygraphy (Table 3). The objective sleep tests have been applied by Jouët et al. (15), Kallwitz et al. (16), Polotsky et al. (17), Agrawal et al. (23), Chen et al. (26), Ng et al. (31), Lian et al. (33), Gul et al. (35), and Trzepizur et al. (36). Nevertheless, there have been some differences in the definition of OSA/SDB as the studies involved different diagnostic thresholds and respiratory variables (e.g., AHI > 10, AHI ≥ 15, RDI ≥ 5, REI ≥ 5, ODI, Tc90%, or minimum oxygen saturation).

**Table 3:** Methodological variables across included studies.

Study ID	Recruitment/sampling	OSA/SDB diagnostic method	OSA severity definition	Liver assessment method	Liver outcome domains	Intervention/exposure contrast	Timing/follow-up	Adjustment/statistical approach
Jouët et al. (15)	Consecutive morbidly obese bariatric candidates	Ambulatory polygraphy	No OSA AHI < 10; moderate 10–50; severe >50	Systematic intraoperative liver biopsy; ALT, AST, GGT	Liver enzymes, steatosis, fibrosis, necroinflammation, MASH	OSA severity groups	Preoperative baseline only	Logistic regression for liver enzymes and MASH; Spearman correlations
Kallwitz et al. (16)	Retrospective review of obesity surgery patients	12-channel PSG	AHI ≥ 15	Intraoperative liver biopsy; ALT/AST	ALT elevation, steatosis, hepatitis, ballooning, fibrosis	OSA vs. non-OSA	Preoperative sleep study and surgery-time biopsy	χ^2^, t-test/Mann–Whitney, logistic regression
Polotsky et al. (17)	Consecutive bariatric candidates recruited prospectively	Full-night sleep study	RDI ≥ 5; hypoxic stress by ΔSaO₂	Liver enzymes in all; biopsy in 20	Insulin resistance, liver enzymes, NAS components, fibrosis	Hypoxemia above vs. below median; RDI severity	Baseline preoperative evaluation	Linear regression and general linear modeling
Shpirer et al. (18)	OSA patients identified in sleep laboratory	In-lab PSG	AHI ≥ 5; mild/moderate/severe OSA	CT liver attenuation index; liver enzymes	Radiologic steatosis; ALT, AST, GGT, ALP	CPAP-compliant vs. noncompliant patients with low LAI	2–3 years CPAP follow-up	Group comparisons using t-test/Fisher exact test
Ulitsky et al. (19)	Retrospective records of bariatric surgery patients	Medical-record OSA diagnosis and/or sleep study	OSA present/absent	Intraoperative liver biopsy	Normal histology, simple steatosis, possible/definite MASH, fibrosis	OSA included as predictor in MASH model	Surgery-time biopsy	Univariate and multivariate logistic regression; AUROC model
Byrne et al. (20)	Consecutive sleep-lab referrals with liver enzymes within 60 days	PSG scored by trained technicians and sleep physician	AHI > 10	ALT and AST only	Biochemical liver injury	OSA vs. no OSA; obese vs. non-obese	Sleep study ±60 days from labs	χ^2^, Fisher exact test, t-tests
Weingarten et al. (21)	Bariatric records with PSG and liver biopsy	Preoperative PSG	AHI ≤ 15 vs. ≥ 16	Intraoperative liver biopsy; AST/ALT	No MASH, mild MASH, advanced MASH; steatosis, fibrosis	No/mild vs. moderate/severe OSA; NIV use	Preoperative PSG and surgery biopsy	Fisher exact, rank-sum, Kruskal–Wallis
Corey et al. (22)	Weight-loss surgery cohort	Medical-record OSA diagnosis	OSA present/absent	Needle liver biopsy at surgery; NAS and fibrosis staging	Normal histology vs. MASLD; NAS; fibrosis	Absence of OSA as protective predictor	Surgery-time biopsy	Logistic regression; ROC model
Agrawal et al. (23)	Consecutive MASLD clinic and OSA clinic patients	Overnight PSG in symptomatic MASLD; PSG-proven OSA cohort	AHI ≥ 5; mild 5–15, moderate 15–30, severe >30	Ultrasound, transient elastography, liver enzymes, biopsy when available	MASLD prevalence, LSM-defined fibrosis, enzymes, histology when available	MASLD-to-OSA and OSA-to-MASLD bidirectional evaluation	Baseline clinic evaluation	Logistic regression for predictors
Jullian-Desayes et al. (24)	Participants from three sham-controlled CPAP RCTs	Overnight sleep study	AHI > 15	FibroMax: SteatoTest, NashTest, FibroTest; liver enzymes	Non-invasive steatosis, MASH, fibrosis biomarkers	Effective CPAP vs. sham CPAP	6, 8, or 12 weeks	ITT and per-protocol analysis; adjusted models
Toyama et al. (25)	Male OSA patients with abdominal CT before and after CPAP	OSA clinical diagnosis with sleep testing	AHI and % sleep time SpO₂ < 90%	CT liver fat content/liver-spleen attenuation; metabolic markers	Liver fat content; fatty liver subgroup response	Long-term CPAP; fatty liver vs. non-fatty liver at baseline	Mean 31 months	Within-group before–after and subgroup analysis
Chen et al. (26)	Consecutive sleep-lab referrals	Full PSG	AHI, ODI, T90%, lowest and average oxygen saturation	Ultrasound steatosis score; ARFI elastography; aminotransferases	Steatosis score, ALT, AST, ARFI fibrosis	OSA severity groups; CPAP before–after subgroup	3 months CPAP in 28 patients	Stepwise regression adjusted for age, sex, smoking, BMI, waist circumference, metabolic markers
Kim et al. (27)	Database-derived OSA patients with ALT before and after CPAP	PSG and CPAP adherence data	AHI; mild/moderate/severe OSA; adherence class	ALT-based suspected MASLD; APRI for fibrosis	ALT, AST, APRI, suspected MASLD regression	CPAP adherence and OSA severity	ALT within 3 months before and within 6 months after CPAP	Multivariable logistic regression adjusted for obesity class and OSA severity
Sundaram et al. (28)	Children from biopsy-proven MASLD cohort with OSA/hypoxia	PSG	AHI, oxygen nadir, nocturnal hypoxia	Baseline liver biopsy; ALT; oxidative stress markers	ALT, metabolic syndrome markers, F2-isoprostanes	Before–after CPAP	Mean 89 ± 62 days CPAP	Within-person comparison
Zhang et al. (29)	Obese patients undergoing metabolic bariatric surgery	PSG before and after surgery	AHI, ODI, oxygenation variables	CT liver/spleen ratio; NFS; liver enzymes	MASLD, steatosis severity, fibrosis score, ALT/AST/GGT	Mild–moderate vs. severe OSA; before–after surgery	6 months after surgery	Correlation analysis and linear regression
Hirono et al. (30)	Consecutive moderate-to-severe OSA patients indicated for CPAP	PSG	AHI > 15; CPAP mean compliance index	Ultrasound MASLD; FibroScan CAP/LSM; serum fibrosis markers	ALT, AST, CAP, LSM, M2BPGi, type IV collagen 7 s	MASLD vs. normal liver; pre/post CPAP in MASLD	6 months CPAP	Univariate and multivariable regression adjusted for body-weight change
Ng et al. (31)	Biopsy-proven MASLD patients screened for OSA	Home sleep test	REI, ODI3, minimum SaO₂, time SaO₂ < 90%	Proton magnetic resonance spectroscopy IHTG; FibroScan CAP/LSM; CK-18; enzymes	IHTG, CAP, LSM, ALT, CK-18	Auto-CPAP vs. subtherapeutic CPAP	6 months	Intention-to-treat; per-protocol; repeated-measures ANOVA; regression
Jawa et al. (32)	Confirmed OSA patients with abdominal ultrasound	PSG	AHI mild/moderate/severe	Ultrasound; ALT; NFS; FIB-4	Suspected MASLD; fibrosis risk	OSA severity categories	Baseline retrospective review	Logistic regression
Lian et al. (33)	Consecutive non-obese suspected sleep apnea patients	Overnight PSG	AHI, ODI, T90%, lowest oxygen saturation	Abdominal ultrasound; FIB-4; liver enzymes	MASLD, liver fibrosis, liver injury	Simple snoring vs. increasing OSA severity	Baseline retrospective analysis	Multivariable logistic regression
Landete et al. (34)	OSA patients and normal lung function controls	Cardiorespiratory polygraphy	AHI, ODI, Tc90%	FLI, HSI, OWLiver lipidomic MASH test, FIB-4, NFS, Hepamet	Hepatosteatosis, MASH profile, advanced fibrosis risk	OSA vs. control; hypoxemia metrics	Baseline only	Multivariable logistic regression
Gul et al. (35)	Adults suspected of OSA	Full-night video PSG	AHI, ODI, mean/minimum oxygen saturation	FibroScan CAP and LSM; FIB-4; liver enzymes	MASLD and fibrosis	OSA severity/hypoxemia indices	Baseline assessment	Multivariable logistic regression
Trzepizur et al. (36)	Suspected MASLD patients referred for percutaneous liver biopsy	In-lab PSG	AHI ≥ 15 for moderate–severe OSA	Liver biopsy using MASH-CRN scoring	Advanced fibrosis *F* ≥ 3; steatosis; MASH	Moderate–severe OSA vs. no/mild OSA	Baseline prospective biopsy/PSG evaluation	Adjusted logistic regression for sex, age, diabetes, obesity

### Liver outcome assessment

Concerning the assessment of liver outcome, a high variability of research methodology can be observed. Biopsy results could be identified in Jouët et al. (15), Kallwitz et al. (16), Weingarten et al. (21), Corey et al. (22), Sundaram et al. (28), and Trzepizur et al. (36). Other studies used ultrasound, computed tomography, FibroScan controlled attenuation parameter (CAP) and liver stiffness measurement (LSM), FibroMax, acoustic radiation force impulse (ARFI) elastography, Fibrosis-4 index (FIB-4), NAFLD fibrosis score (NFS; retained as the original validated score name), aspartate aminotransferase-to-platelet ratio index (APRI), liver-to-spleen ratio, aminotransferases, or lipidomic MASH profiling.

### Statistical adjustment and follow-up

Despite the fact that most cohort studies have applied the regression-based statistical adjustment, the number of confounding factors adjusted for varied. Thus, the stronger statistical adjustment could be found in the cases when body mass index, metabolic syndrome, diabetes, age, and sex had been considered as potential confounders. These include Corey et al. (22), Agrawal et al. (23), Chen et al. (26), Kim et al. (27), Lian et al. (33), Gul et al. (35), and Trzepizur et al. (36). As for follow-up data, they have been provided in the case of the CPAP or bariatric surgery interventions. The follow-up period amounted from 6 to 31 months (24, 25), while other studies were basically cross-sectional assessments of association.

### Steatosis and MASLD burden

**Figure 2:**
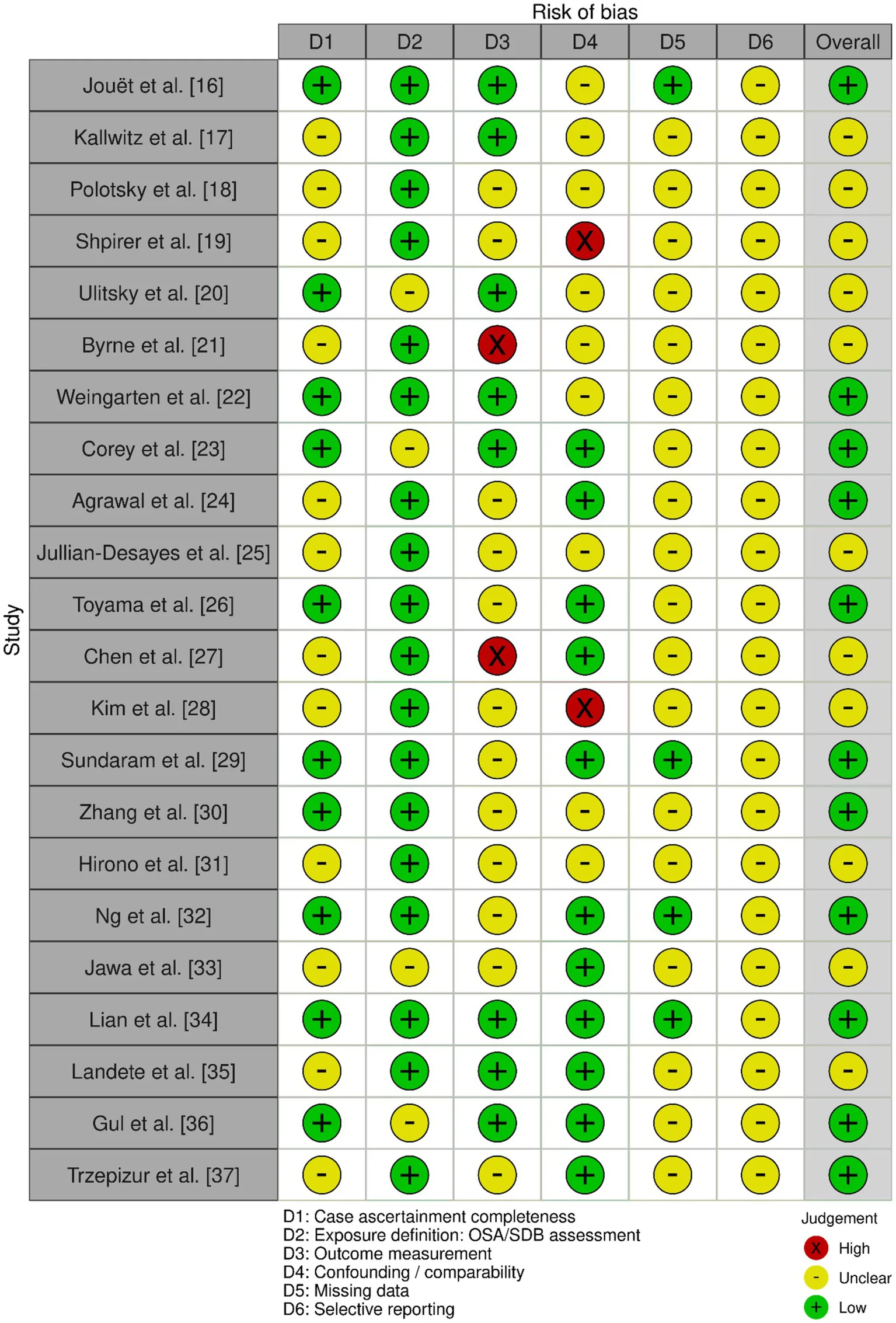
Bias levels observed across the included studies.

The high incidence of steatosis and MASLD was commonly encountered among patients from OSA/SDB-enriched populations (Table 4). The prevalence of steatosis or MASLD was 83.6% in morbidly obese patients according to Jouët et al. (15); 84% in bariatric patients by Weingarten et al. (21); 91.3% in OSA patients by Agrawal et al. (23); 89.4% in suspected MASLD by Kim et al. (27); 65% in non-obese patients with OSA by Lian et al. (33); and 76% in patients with MASLD by Gul et al. (35). Moreover, Chen et al. (26) noted a dose–response relationship between OSA severity and steatosis prevalence. Indeed, the percentage of patients with steatosis was 20.0% in controls, 59.5% in patients with moderate OSA, and 81.8% in patients with severe OSA (Figure 2).

**Table 4:** Outcome-associated variables across included studies.

Study ID	Primary liver outcome	Steatosis result	MASH result	Fibrosis result	Enzyme result	Hypoxia/OSA signal	Effect estimate/statistical result	Treatment/longitudinal finding	Overall inference drawn
Jouët et al. (15)	Liver enzymes and biopsy-defined MASH	Steatosis present in 83.6%	MASH present in 34.4%; similar with and without OSA	Fibrosis present in 54.1%	Elevated enzymes in 46.5%; OSA independently associated with elevated enzymes	AHI not correlated with histologic lesions	OSA and male sex independently predicted elevated liver enzymes; OSA did not predict MASH	No intervention	OSA was related to biochemical liver injury but not to biopsy-defined MASH in morbid obesity.
Kallwitz et al. (16)	ALT elevation and histologic progression	Almost all patients had MASLD	Progressive histology signal only as inflammation plus fibrosis	Fibrosis in 19%; bridging fibrosis/cirrhosis in 5%	ALT elevation more frequent with OSA: 13/39 vs. 3/34, *p* = 0.01	OSA defined by AHI ≥ 15	Trend for OSA in inflammation plus fibrosis: 11/15 vs. 22/48, *p* = 0.06	No intervention	OSA was associated with ALT elevation and showed a borderline histologic progression signal.
Polotsky et al. (17)	Insulin resistance and biopsy MASLD severity	Steatosis not affected by nocturnal hypoxia	Hypoxemia suggested higher hepatic inflammation/ballooning	Hypoxemia suggested fibrosis tendency	Liver enzymes normal overall	Oxygen desaturation >4.6% associated with 1.5-fold higher insulin resistance	RDI was not related to liver histology; hypoxic stress was more informative	No intervention	Hypoxic stress, rather than RDI, appeared linked to occult steatohepatitis biology.
Shpirer et al. (18)	CT liver attenuation index	Low LAI in 16/47; moderate–severe OSA associated with worse LAI	Not assessed by biopsy	Not assessed	ALT, AST, ALP, GGT higher in low-LAI/moderate–severe OSA groups	Moderate–severe OSA had worse LAI despite comparable BMI/triglycerides	CPAP-compliant patients improved LAI; noncompliant patients did not	2–3 years CPAP showed radiologic improvement in compliant patients	Long-term CPAP may improve radiologic steatosis in selected OSA patients with fatty liver.
Ulitsky et al. (19)	Prediction of biopsy-defined MASH	Simple steatosis in 45.8%; normal liver in 33.6%	MASH in 20.6%	Significant fibrosis in 10 patients; 19% of MASH cohort	Abnormal ALT independently predicted MASH	Sleep apnea showed trend and was retained in model	OSA OR 1.86, 95% CI 0.93–3.69, *p* = 0.08; AUROC 0.76 model	No intervention	OSA was not an independent predictor but contributed weakly to a MASH risk model in morbid obesity.
Byrne et al. (20)	Biochemical liver injury	Not directly assessed	Not assessed	Not assessed	ALT elevation: 31% with OSA vs. 6% without OSA, *p* = 0.041; mean ALT higher with OSA	OSA outperformed obesity for ALT association	Mean ALT 30.9 vs. 22.1, *p* = 0.024	No intervention	OSA was associated with biochemical liver injury independent of obesity, but no structural liver outcome was measured.
Weingarten et al. (21)	MASH severity by biopsy	Steatosis in 84%	No MASH 43.1%, mild MASH 43.1%, advanced MASH 13.8%; no association with OSA severity	Fibrotic changes in 34%	No difference in AST/ALT by OSA severity	Moderate/severe OSA had worse PSG oxygenation but not worse histology	MASH category distribution did not differ by OSA severity, *p* = 0.435	NIV prescribed for OSA; no treatment-effect inference	In morbidly obese bariatric patients managed with NIV, OSA severity was not associated with MASH severity.
Corey et al. (22)	Normal liver histology vs. MASLD	110/159 had MASLD; 49 normal	NAS ≥ 5 in 25.5% of MASLD patients	88% of MASLD patients had stage 0–1 fibrosis	Low ALT predicted normal histology	Absence of OSA protective	Absence of OSA OR 5.6, 95% CI 2.0–16.1 for normal histology; model AUROC 0.85	No intervention	Absence of OSA strongly predicted normal liver histology in obese bariatric patients.
Agrawal et al. (23)	MASLD prevalence in OSA and fibrosis in MASLD	MASLD found in 91.3% of OSA patients	Histologic MASH not universal/extractable	AHI independently predicted significant fibrosis	Raised transaminases in 30.4% of OSA patients	AHI was the only significant predictor of fibrosis	AHI OR 1.084, 95% CI 1.002–1.172 for significant fibrosis	No intervention	OSA severity was strongly related to fibrosis risk in an Indian MASLD population.
Jullian-Desayes et al. (24)	Effect of CPAP on non-invasive liver injury markers	Baseline steatosis: 40.4% sham vs. 45.5% CPAP	Borderline/possible MASH: 39.6% sham vs. 58.4% CPAP	FibroTest (serum-based fibrosis component of FibroMax) outcome did not improve	Liver biomarkers did not improve with CPAP	Moderate–severe OSA population; baseline AHI > 15	Effective CPAP did not reduce steatosis, MASH, or fibrosis after adjustment	6–12 weeks CPAP negative versus sham	Short-term effective CPAP did not improve non-invasive MASLD/MASH/fibrosis markers.
Toyama et al. (25)	Long-term CPAP effect on CT liver fat content	Fatty liver in 25/61 at baseline; liver fat decreased in baseline fatty-liver subgroup	Not assessed	Not assessed	Not central/extractable as primary	Baseline fatty liver group had higher AHI and more sleep time SpO₂ < 90%	AHI 52.3 vs. 41.8/h and % sleep time SpO₂ < 90% 30.4 vs. 14.8% in fatty liver vs. non-fatty liver groups	Mean 31 months CPAP; liver fat decreased only in baseline fatty liver subgroup	Long-term CPAP may reduce CT-measured liver fat in male OSA patients with baseline fatty liver.
Chen et al. (26)	Liver steatosis increased across control, moderate OSA, and severe OSA: 20.0, 59.5, and 81.8%	Not assessed histologically	Acoustic radiation force impulse (ARFI) elastography did not differ significantly across groups	ALT and AST increased with OSA severity; ALT 54.20 ± 24.34 to 46.52 ± 24.95 after CPAP; AST 31.82 ± 8.91 to 29.00 ± 8.34	AHI independently predicted steatosis score, ALT, and AST	AHI predicted steatosis score β = 0.447, ALT β = 0.266, AST β = 0.351	3 months CPAP improved ALT and AST without significant BMI change	Supports OSA–enzyme/steatosis link, but fibrosis was not supported	OSA severity was associated with steatosis and biochemical liver injury, and short-term CPAP improved aminotransferases.
Kim et al. (27)	sMASLD present in 89.4%; higher in moderate–severe OSA than mild OSA	Not directly assessed	Aspartate aminotransferase-to-platelet ratio index (APRI) improved after CPAP; significant fibrosis tended to increase with OSA severity	ALT, AST, and APRI improved after CPAP	Greater CPAP adherence showed larger ALT/AST reduction	Good adherence independently predicted sMASLD regression: OR 3.93, 95% CI 1.29–11.94	CPAP >3 months with good adherence was beneficial	Findings are biochemical and database-based, not imaging/biopsy-confirmed	CPAP adherence was associated with biochemical improvement and lower fibrosis-index burden in OSA patients with suspected MASLD.
Sundaram et al. (28)	Baseline biopsy-proven MASLD; no repeat biopsy	Baseline significant MASLD activity; mean NAS 4.8 ± 1.6	Baseline fibrosis stage 1.4 ± 1.3	ALT decreased after CPAP	OSA/hypoxia improved with CPAP	Severe OSA/hypoxia at baseline with elevated aminotransferases and oxidative stress	Mean 89 ± 62 days CPAP reduced ALT, metabolic syndrome markers, and F2-isoprostanes despite BMI increase	Strong mechanistic signal but very small uncontrolled sample	Treating pediatric OSA/nocturnal hypoxia may reduce liver injury and oxidative stress in biopsy-proven pediatric MASLD.
Zhang et al. (29)	MASLD prevalence 75.0% in mild–moderate OSA vs. 96.0% in severe OSA; liver steatosis improved after surgery	Not biopsy-defined	NAFLD fibrosis score (NFS; original validated score name) improved after surgery	ALT, AST, GGT higher in severe OSA at baseline and improved after surgery	OSA metrics correlated with LSR and NFS at baseline	AHI correlated with LSR *r* = −0.219, *p* = 0.003 and NFS *r* = 0.360, *p* < 0.001	Surgery improved OSA, nocturnal hypoxia, steatosis, and fibrosis after 6 months	Surgery-related weight loss is a major confounder	Greater OSA severity was associated with more severe MASLD-related indices at baseline, while bariatric surgery was associated with improvements in both OSA severity and liver-related outcomes.
Hirono et al. (30)	CAP did not significantly improve after CPAP	Not assessed	Liver stiffness measurement (LSM) and serum fibrosis markers did not improve	AST and ALT significantly decreased in MASLD subgroup	CPAP compliance tended to predict ALT improvement independent of weight change	High m-CI tended to improve ALT after adjustment for body-weight change, *p* = 0.051	6 months CPAP improved AST/ALT, especially in compliant patients without BMI change	Suggests enzyme benefit from reoxygenation, but not structural improvement	Effective CPAP improved biochemical liver injury but did not improve steatosis or fibrosis measures over 6 months.
Ng et al. (31)	CAP correlated with OSA severity, but CPAP did not improve CAP vs. control	CK-18 measured; no clear CPAP superiority	Liver stiffness measurement (LSM) did not improve vs. subtherapeutic CPAP	ALT decreased within auto-CPAP group but between-group superiority was not shown	CAP correlated with REI, ODI3, and time SaO₂ < 90%	Weight change correlated with CAP change: B = 3.249, 95% Wald CI 1.538–4.960, *p* < 0.001	Therapeutic and subtherapeutic CPAP had similar effects after 6 months	Weight change mattered more than CPAP assignment	Randomized evidence suggested CPAP alone did not meaningfully alter MASLD status over 6 months.
Jawa et al. (32)	Ultrasound MASLD in 44.4%; suspected MASLD common	Not assessed	High NFS 9%; high FIB-4 3.8%	Elevated liver enzymes in 63.9%	OSA severity was not a fibrosis predictor	Age and BMI predicted high NFS; OSA severity did not	No intervention	Obesity and age explained fibrosis-risk signal more than OSA severity	MASLD was common in OSA, but fibrosis risk was mainly explained by age and BMI.
Lian et al. (33)	MASLD in 228/351 OSA patients, 65%; 56% in simple snoring	Not assessed	Fibrosis in 17/351 OSA patients, 5%; none in simple snoring	ALT and AST increased with OSA aggravation	AHI independently predicted MASLD and fibrosis	AHI predicted MASLD OR 1.02, 95% CI 1.00–1.03; fibrosis OR 1.04, 95% CI 1.00–1.07	No intervention	Important because obesity confounding was reduced	OSA severity was independently associated with MASLD and fibrosis even in non-obese patients.
Landete et al. (34)	Hepatosteatosis more frequent in OSA: FLI 62.1% vs. 34.9%; HSI 68.0% vs. 48.8%	OWLiver lipidomic MASH-risk profile was present in 50.3% of OSA patients vs. 27.9% of controls	Advanced fibrosis rare and not significantly different	Liver enzymes were not the main discriminating endpoint	Tc90% ≥ 10% independently associated with MASH	Tc90% ≥ 10% OR 2.32, 95% CI 1.12–4.81, *p* = 0.023 for MASH	No intervention	Hypoxemia burden was more informative than AHI/ODI alone	Prolonged nocturnal desaturation was independently associated with MASH-risk phenotype in OSA.
Gul et al. (35)	MASLD present in 76%	Not assessed	Fibrosis present in 34.5%	ALT independently associated with fibrosis	Fibrosis group had higher AHI/ODI and lower minimum SpO₂ before adjustment	MASLD: BMI OR 1.09 and metabolic syndrome OR 3.34; fibrosis: BMI OR 1.02, metabolic syndrome OR 2.03, ALT OR 1.02	No intervention	OSA–liver association attenuated after metabolic adjustment	MASLD/fibrosis clustered with OSA severity, but independent drivers were mainly BMI and metabolic syndrome.
Trzepizur et al. (36)	No association reported with steatosis	No association reported with MASH	Advanced fibrosis in 40/97 patients	Not primary	Moderate–severe OSA more common in advanced fibrosis	Moderate–severe OSA: 82% with advanced fibrosis vs. 52% with F0–F2; adjusted OR 3.48, 95% CI 1.03–11.83, *p* = 0.045	No intervention	Strongest fibrosis evidence because biopsy and PSG were used	Moderate–severe OSA was independently associated with biopsy-defined advanced fibrosis in suspected MASLD.

### MASH and fibrosis-related outcomes

The MASH-related outcomes demonstrated a variable association with OSA/SDB. Jouët et al. (15) found the prevalence of MASH equal to 34.4%. However, OSA did not predict biopsy-proven MASH. Weingarten et al. (21) did not find any significant associations between OSA severity and MASH categories (*p* = 0.435). Ulitsky et al. (19) noted only a modest role of OSA in MASH prediction, and in multivariable analysis, there was no significant association (OR 1.86, 95% CI 0.93–3.69; *p* = 0.08).

On the other hand, fibrosis-related outcomes demonstrated a more meaningful association with OSA. Specifically, Agrawal et al. (23) found that AHI was independently associated with significant fibrosis (OR 1.084, 95% CI 1.002–1.172). Lian et al. (33) also noted an association between higher AHI and the risk of fibrosis in non-obese patients with OSA (OR 1.04, 95% CI 1.00–1.07). Furthermore, Trzepizur et al. (36) noted an independent association between moderate-to-severe OSA and biopsy-confirmed advanced fibrosis (OR 3.48, 95% CI 1.03–11.83; *p* = 0.045).

### Liver enzymes and intervention-related outcomes

The opposite pattern was observed for the biochemical markers of liver injury. Specifically, Kallwitz et al. (16) noted significantly higher rates of ALT elevation in patients with OSA as compared with those without OSA (13/39 vs. 3/34; *p* = 0.01). Byrne et al. (20) found the prevalence of ALT elevation higher in patients with OSA compared with those without OSA (31% vs. 6%; *p* = 0.041). The effects of CPAP therapy were mostly seen in the biochemical outcomes. Chen et al. (26) showed a decrease in ALT levels from 54.20 ± 24.34 to 46.52 ± 24.95 U/L and AST levels from 31.82 ± 8.91 to 29.00 ± 8.34 U/L under the CPAP therapy for 3 months. Additionally, Kim et al. (27) reported independent prediction of regression of suspected MASLD with higher CPAP adherence (OR 3.93, 95% CI 1.29–11.94). Nevertheless, randomized and controlled studies performed by Jullian-Desayes et al. (24) and Ng et al. (31) did not show significant improvements in steatosis, MASH, or fibrosis-related parameters.

### Meta-analytic observations

**Figure 3:**
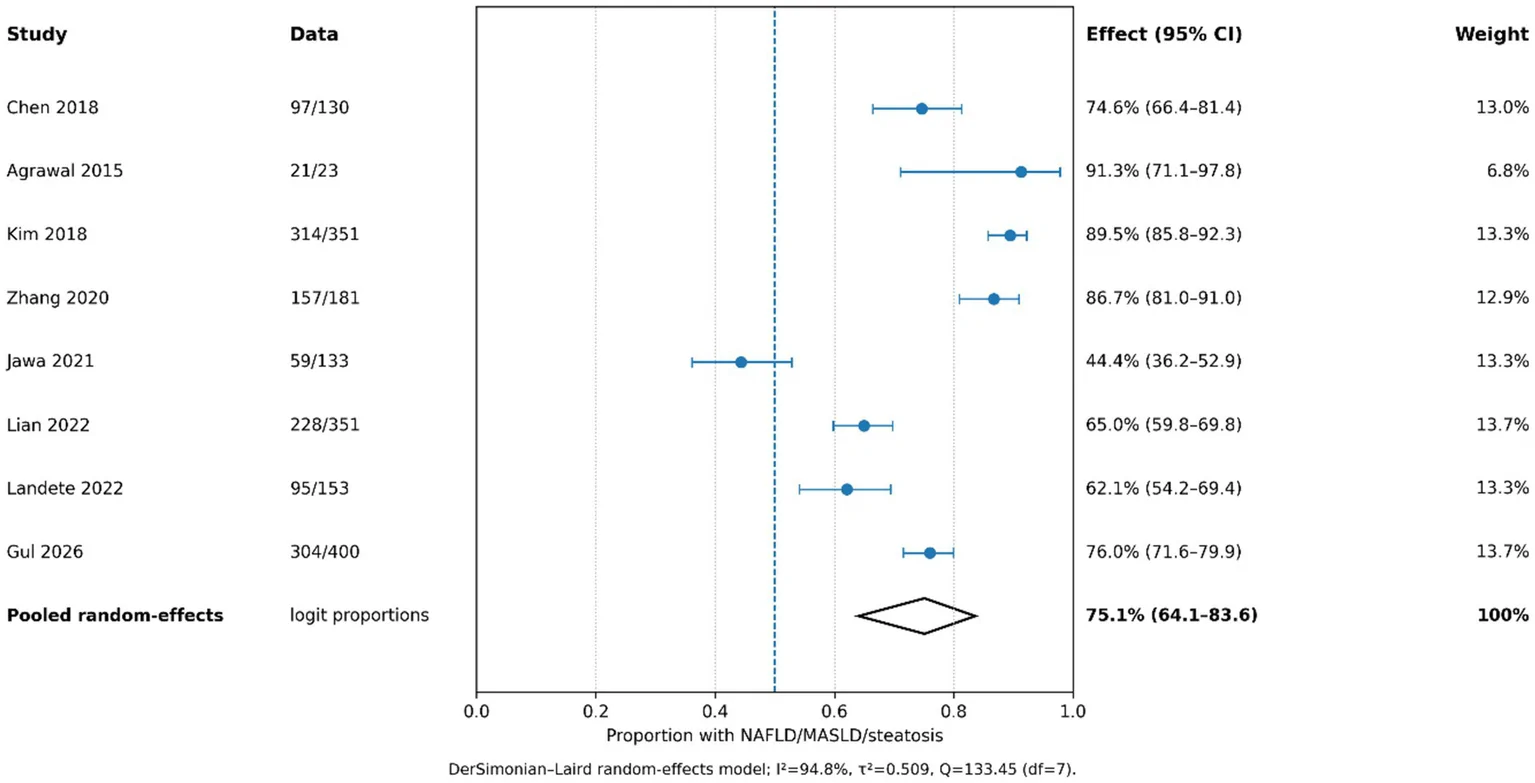
MASLD or steatosis prevalence among patients with sleep-disordered breathing.

As depicted in Figure 3, the prevalence-based meta-analysis indicated a significant MASLD and hepatic steatosis burden in patients with OSA/SDB. The prevalence-based analysis using the random effects model estimated from the eight studies indicated a 75.1% (95% CI 64.1–83.6%) prevalence rate, suggesting that more than three-quarters of patients with sleep-disordered breathing had steatotic liver disease. However, there was significant heterogeneity between studies (I^2^ = 94.8%), meaning that there was a large variation in the prevalence rates. This heterogeneity was probably due to the difference in the method used for assessing liver disease, including steatosis by ultrasound, steatosis by CT, MASLD by CAP, steatosis by FLI, and suspected MASLD by ALT. Nonetheless, majority of individual studies had prevalence estimates above 60%.

**Figure 4:**
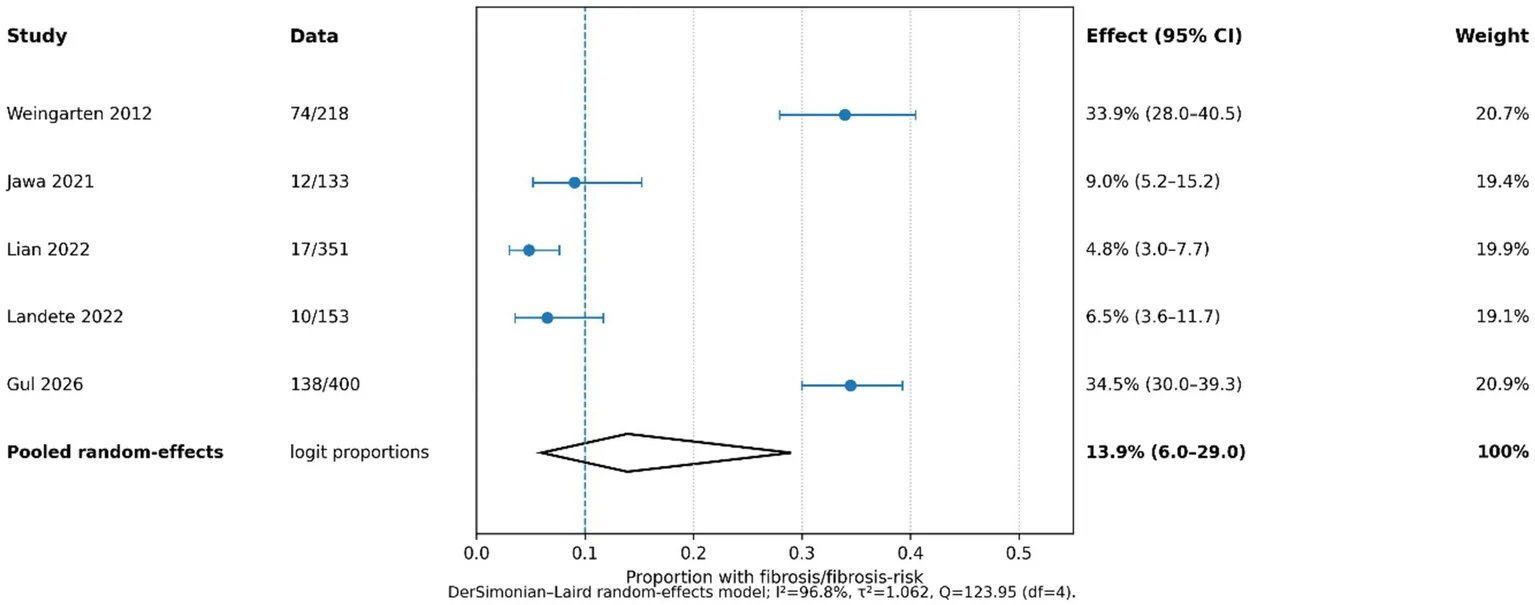
Liver fibrosis or fibrosis-risk prevalence among patients with sleep-disordered breathing.

The prevalence based pooled analysis presented in Figure 4 showed a relatively lower but still significant level of fibrosis in the group of patients with SDB. With the inclusion of five studies, the random effects pooled prevalence of fibrosis/fibrosis risk was 13.9% (95%CI 6.0–29.0%). The wide confidence interval is indicative of a large degree of uncertainty associated with the estimated prevalence of fibrosis, probably due to the variation in the measurement of the presence of fibrosis between the studies, which included biopsy proven fibrosis, NAFLD fibrosis score (NFS), FIB-4 and transient elastography derived fibrosis or liver stiffness. Moreover, there was a high level of heterogeneity among studies (I^2^ = 96.8%).

**Figure 5:**
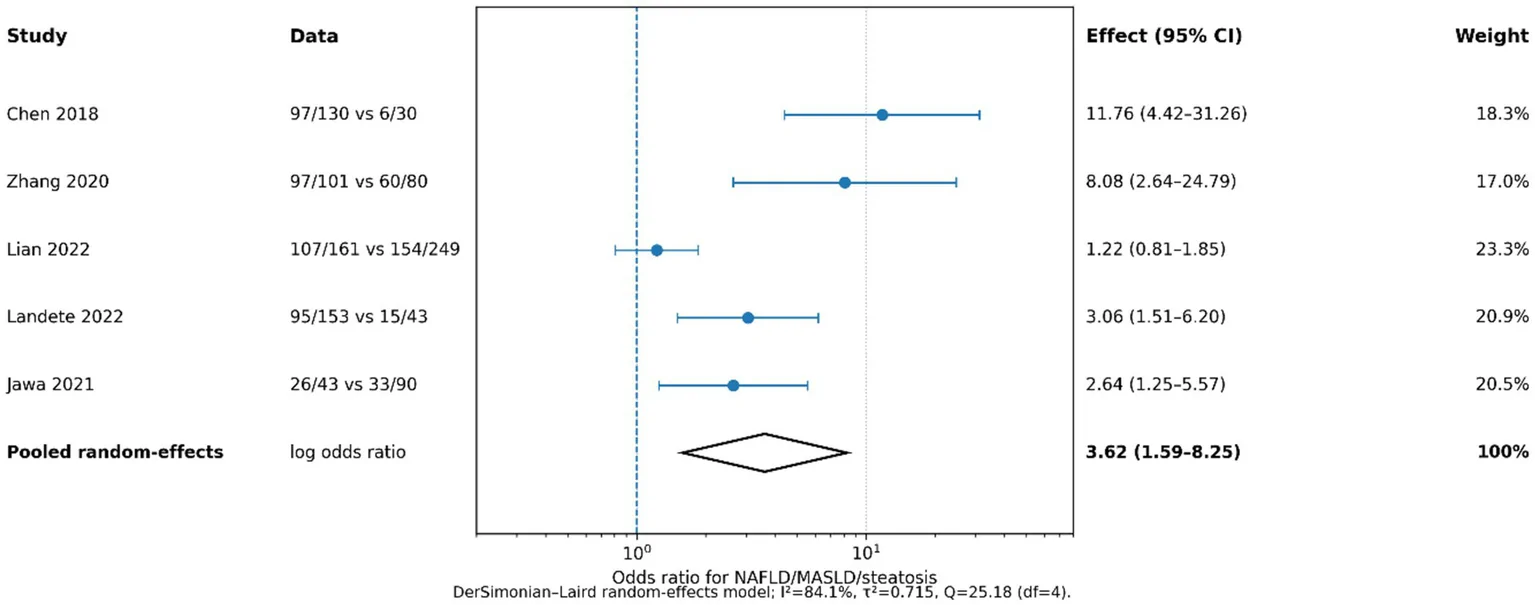
Association between sleep-disordered breathing severity/presence and MASLD/steatosis risk.

As can be seen in Figure 5, a significant odds ratio-based pooled analysis showed an association between SDB and MASLD/steatosis. According to five studies, the random effects model pooled odds ratio was 3.62 with 95% confidence interval from 1.59 to 8.25. As the confidence interval was not equal to 1, there was a statistically significant association between SDB exposure and steatotic liver disease (z-value = 3.06; *p* ≈ 0.002), meaning that the presence of OSA/SDB or the higher severity of OSA was linked to the three times higher odds of MASLD/steatosis. Nevertheless, the high heterogeneity (I^2^ = 84.1%) indicated the variance of the effect size across studies due to the different OSA classification, control group definition, obesity frequency and liver outcome definitions. Still, the effect direction was generally positive meaning that the burden of steatotic liver disease grew according to OSA severity. This effect estimation should be interpreted with caution due to the following two reasons. The pooled analysis included both studies where the comparison was made between OSA presence vs. absence and studies where the comparison was performed regarding the severity of OSA which are conceptually different and were not separated in the analysis. Additionally, the data included in the analysis was not equally adjusted: the 2 × 2 numbers used for the pooled OR estimation (6/30 in the control group vs. 97/130 in the combined moderate-to-severe OSA group) in the case of Chen et al. (26) represent the unadjusted numbers by severity groups, and not the covariate adjusted effects, as the original article presented only standardized regression coefficients for continuous AHI and not adjusted odds ratios for this specific comparison. Therefore, the pooled OR of 3.62 is the mixture of adjusted/unadjusted, presence/severity comparisons and, considering the very high heterogeneity (I^2^ = 84.1%) and confidence interval, is a hypothesis-generating association.

**Figure 6:**
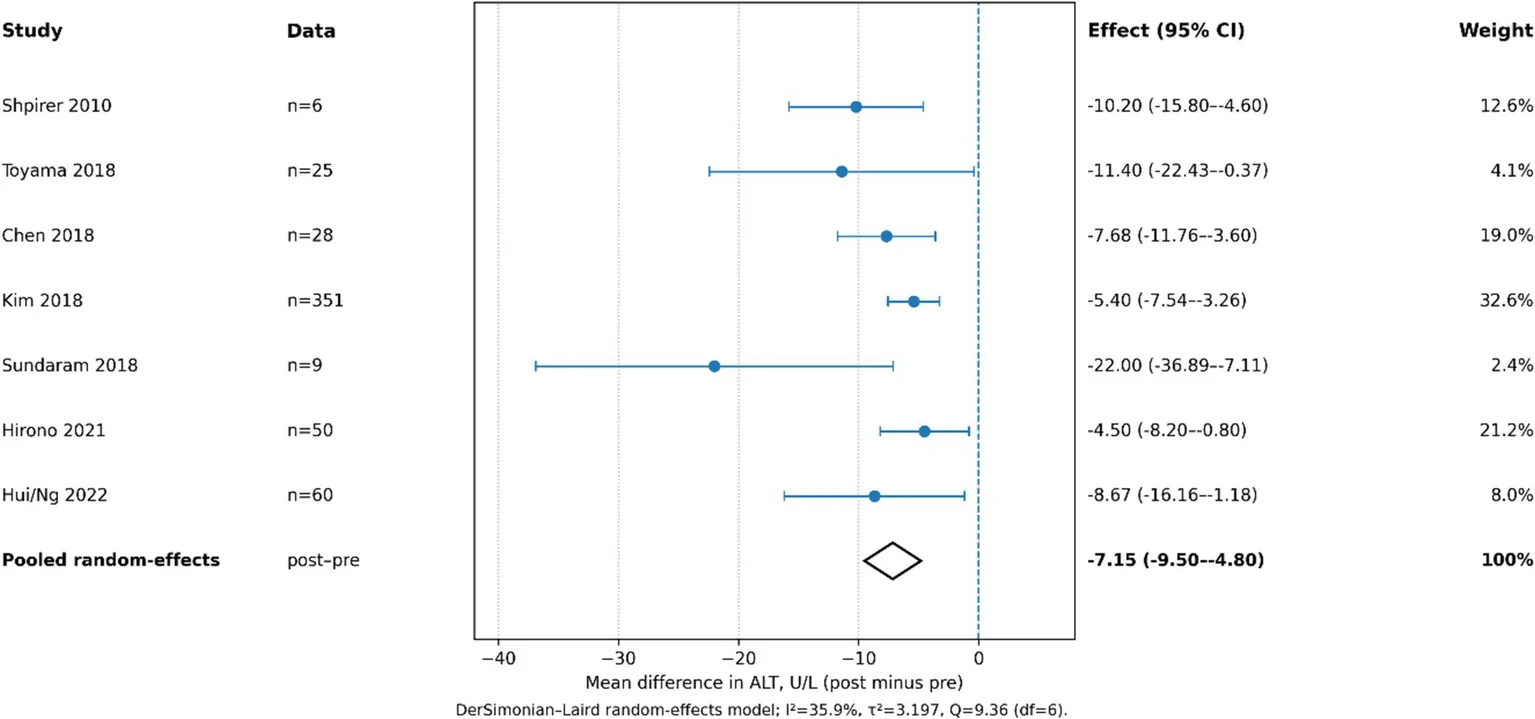
Change in alanine aminotransferase after continuous positive airway pressure therapy.

As depicted in Figure 6, the pooled analysis for CPAP studies revealed statistically significant reduction in ALT levels. On the basis of the results of seven studies, the pooled mean difference was found to be −7.15 U/L, with the 95% confidence interval ranging from −9.50 to −4.80 U/L. As the confidence interval was below zero, the difference was thus statistically significant (z-value = −5.96, *p* < 0.001). Meanwhile, low-to-moderate heterogeneity (I^2^ = 35.9%) indicated low variability in the results of ALT changes as compared to the prevalence and odds ratio analyses. The greatest decrease in ALT level was reported for small before-and-after CPAP populations or those with fatty liver, whereas lower decreases were observed in database- and randomized trials-derived samples.

**Figure 7:**
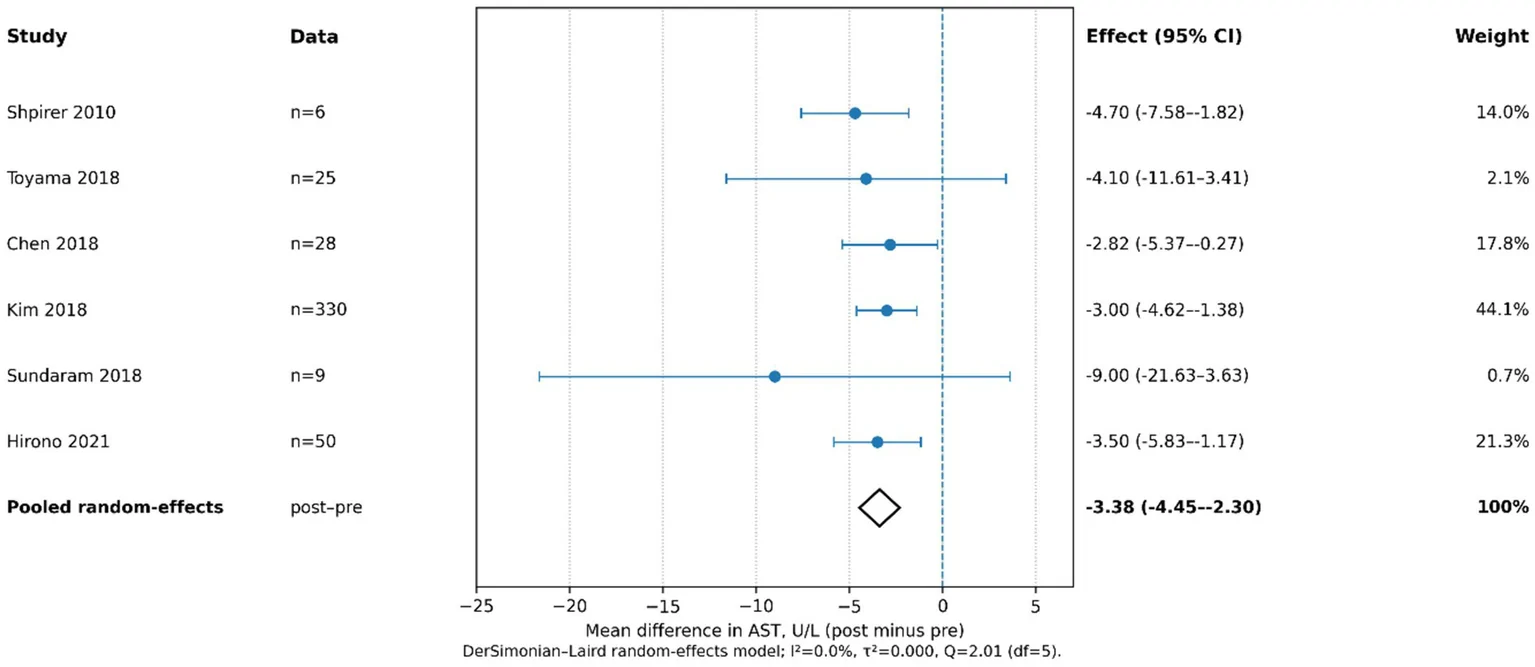
Change in aspartate aminotransferase after continuous positive airway pressure therapy.

As depicted in Figure 7, the meta-analysis of AST based on the six articles showed a statistically significant result. Based on 6 articles, the random effects pooled mean difference was −3.38 U/L, and the 95% confidence interval was between −4.45 and −2.30 U/L. Confidence interval implies the presence of reliable reduction of AST (z-value = −6.17, *p* < 0.001). In addition, it was observed that the heterogeneity obtained is small (I^2^ = 0%). This is due to the fact that there was high consistency of the decrease in AST regardless of the variability in terms of population age, presence of liver disease, use of CPAP therapy, and the type of study. According to Kim et al. (27), the analytical denominator for AST (*n* = 330) was lower compared to that of ALT (*n* = 351, Figure 6). This is not due to any mistake in the size of the total cohort provided in Table 2 but the availability of data of AST before-and-after comparison.

### Certainty assessment observations

Certainty assessment showed that the majority of outcome domains were characterized by a very low and low certainty of evidence (Tables 5, 6). Despite the high prevalence of MASLD or steatosis in people with OSA/SDB, there was one important limitation concerning heterogeneity of liver outcome definition (e.g., ultrasound, CT, fibroscan CAP, FLI, and ALT-based suspicion of MASLD) (23, 26, 27, 29, 32–35). On the other hand, pooled exposure to OSA/SBD exposure was positively correlated with the risk of steatosis; however, the same problem related to the high heterogeneity of outcomes made certainty of the evidence very low (26, 29, 32–34).

**Table 5:** GRADE evidence profile.

Evidence domain	No. of studies	Evidence base	Bias concerns	Consistency of findings	Indirectness	Precision of estimate	Reporting bias concern	Final certainty
MASLD or steatosis burden among OSA/SDB patients (23, 26, 27, 29, 32–35)	8	Mostly observational cohorts, sleep-clinic cohorts, bariatric cohorts	Serious	Very serious; I^2^ was 94.8%	Serious; different liver definitions were used	Not serious; large total sample	Unclear	Very low
Association between OSA/SDB severity or presence and MASLD/steatosis risk (26, 29, 32–34)	5	Observational comparative cohorts	Serious	Serious; I^2^ was 84.1%	Serious; exposure contrasts differed by study	Serious; confidence intervals were wide	Unclear	Very low
Fibrosis or fibrosis-risk prevalence in OSA/SDB cohorts (21, 32–35)	5	Observational cohorts	Serious	Very serious; I^2^ was 96.8%	Serious; fibrosis was measured by biopsy, LSM, NFS, or FIB-4	Serious; pooled CI was wide	Unclear	Very low
OSA/SDB severity and liver fibrosis/advanced fibrosis association (23, 33, 35, 36)	4	Observational cohorts, including biopsy-based and non-invasive fibrosis studies	Serious	Serious; direction was not uniform across studies	Moderate; outcome methods varied, but fibrosis was clinically relevant	Serious; several estimates had wide CIs	Unclear	Low
CPAP therapy and ALT reduction (18, 25–28, 30, 31)	7	Mixed interventional evidence, mainly before–after CPAP cohorts with one randomized CPAP trial	Serious	Not serious; I^2^ was 35.9%	Serious; ALT was a biochemical surrogate	Not serious; pooled CI excluded null	Unclear	Low
CPAP therapy and AST reduction (18, 25–28, 30)	6	Mostly non-randomized before–after CPAP cohorts	Serious	Not serious; I^2^ was 0.0%	Serious; AST was a biochemical surrogate	Not serious; pooled CI excluded null	Unclear	Low
CPAP therapy and structural liver outcomes, including steatosis, CAP, LSM, IHTG, or fibrosis markers (18, 24, 25, 29–31)	6	Mixed CPAP and bariatric/interventional cohorts, including randomized CPAP evidence	Serious	Serious; biochemical and structural outcomes diverged	Serious; imaging and non-invasive markers differed	Serious; effects were inconsistent and often non-significant	Unclear	Very low

**Table 6:** Summary of findings.

Outcome assessed	Expected absolute effect	Comparative or pooled effect	Evidence size	GRADE certainty
MASLD or steatosis prevalence among OSA/SDB patients (23, 26, 27, 29, 32–35)	The pooled prevalence was **75.1%**; 95% CI **64.1–83.6%**	Not applicable; single-arm pooled prevalence	Participants from 8 studies	Very low ⊕⊝⊝⊝
Fibrosis or fibrosis-risk prevalence among OSA/SDB patients (21, 32–35)	The pooled prevalence was **13.9%**; 95% CI **6.0–29.0%**	Not applicable; single-arm pooled prevalence	Participants from 5 studies	Very low ⊕⊝⊝⊝
MASLD/steatosis risk in higher OSA/SDB exposure groups (26, 29, 32–34)	Higher OSA/SDB exposure groups had more frequent steatosis/MASLD than comparator groups	Pooled OR **3.62**; 95% CI **1.59–8.25**	Participants from 5 studies	Very low ⊕⊝⊝⊝
Liver fibrosis or advanced fibrosis associated with OSA/SDB severity (23, 33, 35, 36)	Fibrosis risk was higher in selected studies with greater AHI or moderate-to-severe OSA	AHI predicted fibrosis in (23, 33); moderate-to-severe OSA predicted advanced fibrosis in (36) with OR **3.48**, 95% CI **1.03–11.83**	Participants from 4 studies	Low ⊕ ⊕ ⊝⊝
ALT change after CPAP therapy (18, 25–28, 30, 31)	ALT decreased after CPAP in pooled before–after analysis	Mean difference **−7.15 U/L**; 95% CI **−9.50 to −4.80**	Participants from 7 studies	Low ⊕ ⊕ ⊝⊝
AST change after CPAP therapy (18, 25–28, 30)	AST decreased after CPAP in pooled before–after analysis	Mean difference **−3.38 U/L**; 95% CI **−4.45 to −2.30**	Participants from 6 studies	Low ⊕ ⊕ ⊝⊝
Structural liver response after CPAP or related intervention (18, 24, 25, 29–31)	Structural outcomes were inconsistent; some studies showed radiologic or post-bariatric improvement, while controlled CPAP studies did not show clear benefit	No consistent pooled structural effect was established	Participants from 6 studies	Very low ⊕⊝⊝⊝

Bold values indicate the principal pooled effect estimates/findings.

Evidence on fibrosis burden appeared to be somewhat more important but still heterogeneous and limited by heterogeneity of outcomes. In particular, the use of non-invasive score systems, elastography, and even advanced fibrosis with biopsies was reported (32–35) and in Trzepizur et al. study (36). While the direction of effect was clearly associated with increased fibrosis burden with increasing severity of OSA and hypoxemia, there were precision problems in relation to the adjusted fibrosis burden. As a result, certainty in relation to fibrosis burden was rated as low, not high.

In terms of biochemical outcomes associated with CPAP therapy, the results were quite consistent and showed a significant reduction in ALT and AST, having a pooled mean difference of −7.15 U/L and −3.38 U/L, respectively (18, 25–28, 30, 31). At the same time, it should be stressed that there was low certainty of the evidence since almost all studies were non-randomized before-after designs and aminotransferases were surrogate markers. Concerning structural liver outcomes following CPAP treatment, only controlled short-term treatment showed no improvement (24, 31); however, some uncontrolled or longer term ones suggested improvement in selected subgroups (18, 25, 30).

## Discussion

The primary observations drawn from the review include the high prevalence of steatosis in patients with sleep-disordered breathing, high odds of development of MASLD and the presence of mixed results related to fibrosis-related liver outcomes in patients with SDB, the latter being highly dependent on comorbid metabolic conditions. The pooled prevalence of MASLD or steatosis was estimated at 75.1% (95% CI 64.1–83.6%). In turn, the odds of developing steatosis or MASLD were estimated to be more than three times higher in patients with OSA/SDB compared to non-SDB controls (OR 3.62, 95% CI 1.59–8.25). Despite high heterogeneity, the observed associations with a positive direction were noted in bariatric, hepatology, sleep-clinic, and non-obese patient cohorts, thus proving that the association between SDB and steatotic liver disease exists in a broader range of heterogeneous patient populations. Importantly, the present results confirm the growing hypothesis that intermittent hypoxia can lead to liver steatosis independently from other metabolic risk factors. However, the complete separation of obesity-related confounding factors seems impossible in the context of the reviewed literature, the issue which was also emphasized in previous narrative reviews on this topic (37, 38). In contrast to previous literature reviews carried out before 2023 MASLD nomenclature revision, the present review includes not only the historical NAFLD/NASH terminology but also the current terminology for MASLD. In addition, the present review includes a quantitative synthesis of the effectiveness of CPAP therapy in terms of both biochemical and histological liver outcomes. The latter type of data synthesis is absent in previous literature reviews.

The associations revealed seem reasonable and consistent with modern knowledge about liver injury caused by intermittent hypoxia. According to the available experimental and translational literature, nocturnal hypoxemia may contribute to hepatic lipid accumulation via oxidative stress, mitochondrial dysfunction, disruption of lipid metabolism, inflammatory cytokines’ activity and stellate cells stimulation (39). In addition, the contribution of intermittent hypoxia into development of insulin resistance, adipokines regulation disorders, lipotoxicity and chronic low-grade inflammation, all of which are involved in the pathophysiology of MASLD, is well-established (40). Importantly, the current review suggests that hypoxemia indices rather than apnea frequency seem to be more strongly associated with liver injury. Several studies included in this review demonstrated stronger associations with oxygen desaturation index, Tc90% and minimum oxygen saturation as compared to AHI, which confirms the hypothesis about the clinical relevance of hypoxic burden as opposed to apnea frequency in terms of hepatic risk (41). The finding adds valuable information regarding the issue of OSA-related liver injury and can explain some discrepancies between the studies focused on AHI-based severity categories.

The combined analysis of adult and pediatric populations in this meta-review was justified as there was no need to consider hypoxic hepatic injury age-specific and the body of evidence in the latter age group was too scarce to allow conducting of separate synthesis. Specifically, the only pediatric paper (28) (n = 9) made contribution solely into analysis of ALT and AST lowering effect of CPAP and did not provide any information to estimate the prevalence, associations and fibrosis in the review, all of which were assessed in adult cohort exclusively. Notwithstanding the paucity of data, the pediatric studies found similar to adult population in terms of relationships between OSA, increased aminotransferases and signs of liver damage. This pattern is consistent with previous meta-analytic evidence in the pediatric population linking the sleep disorders and MASLD development and abnormalities related to liver fibrosis (42).

Unlike steatotic-related outcomes, the findings of current analysis regarding fibrotic-related liver abnormalities proved to be less consistent and seemed to depend more on the underlying risk factors. Thus, the pooled prevalence of fibrosis/fibrosis-risk reached 13.9% (95% CI 6.0–29.0%) indicating the presence of clinically significant burden of fibrosis among patients with SDB; however, considerable heterogeneity was reported due to differences in fibrosis assessment tools used in the included studies (liver biopsy, transient elastography, FIB-4, NAFLD fibrosis score (NFS) and APRI). It should be noted that the association of OSA severity or hypoxemia burden with fibrosis-related outcome was reported independently in several studies mostly in biopsy-based and non-obese cohorts showing that SDB can contribute to fibrosis progression independently of obesity alone. On the other hand, this review illustrates the problem in the differentiation of the impact of OSA from other cardiometabolic factors (obesity, insulin resistance, diabetes mellitus and chronic systemic inflammation). Thus, the current data support the idea that OSA may act as the disease-modifying factor amplifying metabolic liver injury.

The findings of the current meta-review concerning the treatment of SDB demonstrate the distinction between biochemical and structural liver damage. Indeed, CPAP was shown to be consistently associated with the reduction in the level of aminotransferases (ALT – 7.15 U/L, AST – 3.38 U/L), thus, the elimination of nocturnal hypoxemia seems to be effective in improving the markers of liver injury. At the same time, the evidence about the positive effect on steatosis, MASH activity, liver stiffness and fibrosis-related outcome remains scarce and inconsistent in controlled studies. Such situation rather demonstrates important methodological limitation of existing studies (short follow-up period, poor compliance of patients to the therapy, small sample size and heterogeneity in assessment of structural abnormalities) than the lack of efficacy of CPAP. At the same time, the current analysis showed the difference in the timing of biochemical and structural changes, as the former seem to appear earlier than the latter. Such assumption is confirmed by obesity-related researches that indicate parallel improvement in both OSA and MASLD after the bariatric surgery (43) and demonstrate close interaction of these two pathological processes.

The current analysis emphasizes the cardiometabolic complexity in the relationship between OSA and steatotic liver disease. Fragmentation of sleep, intermittent hypoxia, obesity, insulin resistance, metabolic syndrome and chronic systemic inflammation often coexist and interact synergistically rather than being isolated pathogenic processes (44). Thus, the relationship of OSA and MASLD is likely to be bidirectional and multifactorial. This assumption is confirmed by several Mendelian randomization studies that failed to prove the causality of the effects of sleep apnea on MASLD development, liver enzymes and insulin resistance despite high association (45). Thus, while intermittent hypoxia still can be considered a biologically plausible factor contributing to the liver injury, obesity and metabolic dysfunction remain the main factors of the disease and therapeutic target (38). However, the mechanistic studies confirm the biological plausibility of OSA-related liver injury by demonstrating activation of oxidative stress pathways, inflammatory cytokines, Kupffer cells, lipogenesis and fibrogenic signaling cascades (46).

The clinical relevance of the findings could prove particularly significant in metabolically vulnerable subpopulations. PCOS female patients might be especially prone to the comorbidity of OSA and MASLD due to their similar underlying mechanisms including insulin resistance, hyperandrogenism, obesity, and cardiometabolic dysfunction (47). Large administrative database studies also found significantly increased odds of MASH in patients with OSA taking into account major confounders (48). While such studies still suffer from cross-sectional design and methodology based on coding, they further support the general direction of the associations detected in this review.

### Limitations

A few limitations should be considered in relation to these findings. Heterogeneity between the prevalence and association estimates was high to very high (I^2^ 84.1–96.8%) and the current review did not conduct prespecified subgroup analysis, meta-regression, and leave-one-out sensitivity analysis aimed at the detection of sources of heterogeneity; the analysis of heterogeneity presented above cannot serve as a replacement for quantitative methods, and pooled point estimates should be viewed as indicative rather than precise. Also, the prevalence, association, and fibrosis analyses combined different definitions of outcomes which are not interchangeable in terms of their clinical meaning and included ultrasound, computed tomography, controlled attenuation parameter, fatty liver index, and ALT-based “suspected MASLD” in case of steatosis and biopsy, liver stiffness measurement, FIB-4, NAFLD Fibrosis Score, and APRI in case of fibrosis; the use of ALT-based case definition in particular represents a poor surrogate for MASLD and its use as a part of the primary pooled prevalence estimate rather than stratification likely contributes to heterogeneity. Additionally, publication bias was not formally assessed; funnel plots and Egger’s test were not conducted due to fewer than ten studies contributing to any single pooled outcome below the threshold of reliability for these tests.

### Recommendations and future directions

These findings support the consideration of liver assessment in OSA/SDB patients, especially in the presence of obesity, metabolic syndrome, diabetes mellitus, elevated aminotransferases, or significant nocturnal hypoxemia. The patients with MASLD with clinical characteristics suspicious for underlying sleep apnea should also be screened for sleep disorders. Importantly, the parameters of nocturnal oxygen desaturation appear to correlate better with liver damage than the apnea frequency and might represent a clinically more relevant risk factor for liver damage than AHI alone.

CPAP treatment is associated with improvement in liver damage biochemical markers, but there is insufficient evidence regarding structural liver improvement. This highlights the necessity for comprehensive cardiometabolic management strategies incorporating treatment of sleep-disordered breathing and weight loss. Future studies should concentrate on prospective cohort and randomized trials using standardized sleep measurements, hypoxemia assessment, validated liver outcomes, and long-term follow-up. Further research in non-obese, pediatric and combination therapies populations is needed as well.

## Conclusion

Sleep-disordered breathing is associated with a significant burden of steatosis and increased odds of MASLD in various populations. It is noteworthy that hypoxemia-related parameters appear to be more consistently associated with liver injury than apnea frequency, indicating the potential role of the former as a factor of MASLD development and progression. Although the associations with steatosis are relatively consistent in direction, those with fibrosis-related outcomes are more heterogeneous and depend on metabolic function impairment. CPAP treatment is associated with improvements in biochemical markers of liver injury, but the evidence regarding structural liver changes is insufficient. These findings highlight the sleep-disordered breathing as an important factor modifying metabolic liver disease and call for further integrated cardiometabolic management.

## Data Availability

The original contributions presented in the study are included in the article/supplementary material, further inquiries can be directed to the corresponding author.
